# The *dam replacing gene* product enhances *Neisseria gonorrhoeae* FA1090 viability and biofilm formation

**DOI:** 10.3389/fmicb.2014.00712

**Published:** 2014-12-17

**Authors:** Agnieszka Kwiatek, Pawel Bacal, Adrian Wasiluk, Anastasiya Trybunko, Monika Adamczyk-Poplawska

**Affiliations:** ^1^Department of Virology, Institute of Microbiology, Faculty of Biology, University of WarsawWarsaw, Poland; ^2^Laboratory of Theory and Applications of Electrodes, Faculty of Chemistry, University of WarsawWarsaw, Poland

**Keywords:** *dam replacing gene*, Dam activity, *Neisseria gonorrhoeae*, adhesion, biofilm

## Abstract

Many *Neisseriaceae* do not exhibit Dam methyltransferase activity and, instead of the *dam* gene, possess *drg* (*dam replacing gene*) inserted in the *leuS/dam* locus. The *drg* locus in *Neisseria gonorrhoeae* FA1090 has a lower GC-pairs content (40.5%) compared to the whole genome of *N. gonorrhoeae* FA1090 (52%). The gonococcal *drg* gene encodes a DNA endonuclease Drg, with GmeATC specificity. Disruption of *drg* or insertion of the *dam* gene in gonococcal genome changes the level of expression of genes as shown by transcriptome analysis. For the *drg*-deficient *N. gonorrhoeae* mutant, a total of 195 (8.94% of the total gene pool) genes exhibited an altered expression compared to the wt strain by at least 1.5 fold. In *dam*-expressing *N. gonorrhoeae* mutant, the expression of 240 genes (11% of total genes) was deregulated. Most of these deregulated genes were involved in translation, DNA repair, membrane biogenesis and energy production as shown by cluster of orthologous group analysis. *In vivo*, the inactivation of *drg* gene causes the decrease of the number of live neisserial cells and long lag phase of growth. The insertion of *dam* gene instead of *drg* locus restores cell viability. We have also shown that presence of the *drg* gene product is important for *N. gonorrhoeae* FA1090 in adhesion, including human epithelial cells, and biofilm formation. Biofilm produced by *drg*-deficient strain is formed by more dispersed cells, compared to this one formed by parental strain as shown by scanning electron and confocal microscopy. Also adherence assays show a significantly smaller biomass of formed biofilm (OD_570_ = 0.242 ± 0.038) for *drg*-deficient strain, compared to wild-type strain (OD_570_ = 0.378 ± 0.057). *Dam*-expressing gonococcal cells produce slightly weaker biofilm with cells embedded in an extracellular matrix. This strain has also a five times reduced ability for adhesion to human epithelial cells. In this context, the presence of Drg is more advantageous for *N. gonorrhoeae* biology than Dam presence.

## Introduction

*Neisseria gonorrhoeae* (gonococcus) is the causative agent of a sexually transmitted disease: gonorrhea. Nowadays, gonococcal infection is a global health problem and more than 88 million cases are annually reported worldwide (WHO, 2011).

Susceptibility to chronic complications associated with *N. gonorrhoeae* infection is a specific problem for women due to asymptomatic cervicitis and subsequent spread of gonococcus to the upper genital tract. Ascending infection occurs in up to 45% of infected women and may result in the pelvic inflammatory disease, which can cause permanent fallopian tube scarring and blockage, with subsequent infertility and ectopic pregnancies (Holmes, 1999). Furthermore, it was demonstrated that in response to gonococcal infection, genital epithelial cells produce cytokines, chemokines and defensins that can modulate the human immunodeficiency virus (HIV) infection and infectivity (Jarvis and Chang, 2012).

Gonococcal pathogenicity is tightly connected with the ability to form biofilm. Several studies demonstrate that *N. gonorrhoeae* can attach to different surfaces (glass, human cervical cells or in continuous flow-chamber systems) and form biofilms (Greiner et al., 2005; Falsetta et al., 2011; Zweig et al., 2014). Moreover, it was demonstrated that *N. gonorrhoeae* can also form biofilm structures within the human female genital tract (Steichen et al., 2008). Gonococcal biofilms, as part of cervical infection, may be involved in mechanisms by which asymptomatic infections, persistence, and increased antibiotic resistance occur.

One of the factors causing *N. gonorrhoeae* pathogenesis is its phase variation connected to hetero- and homopolymeric tracts (Broadbent et al., 2010; Srikhanta et al., 2010). The length of these tracts may change, which can have a high impact on gene expression (Snyder et al., 2001; Jordan et al., 2005). Mutations that occur within such sequences during replication of chromosomal DNA, may lead to changes in the reading frame (frameshifts) or incorrect pairing of nucleotides (mismatches). This is due to the slippage of DNA polymerase III, termed slipped-strand misparing (SSM). It has been shown that the frequency of phase variation due to SSM is regulated by the Dam methylase (MTase) in such bacteria as *Escherichia coli, Haemophilus influenzae*, or *Salmonella* (Bucci et al., 1999; Zaleski et al., 2005; Broadbent et al., 2010). This regulation is absent in gonococcal and meningococcal *dam*-deficient strains.

Dam MTase is not only engaged in the regulation of phase variation, but is also the key protein of the methyl-directed mismatch repair system (MMR), which is involved in repair of mutations resulting from incorrectly paired nucleotides. The MMR system has been thoroughly studied in *E. coli* and it was shown to be directed by DNA methylation by Dam MTase, which marks the parental strand (methylation of adenine in the GATC sequence). This allows the unmethylated DNA daughter strand repair (Marinus and Casadesus, 2009). Such regulatory mechanism occurs at bacterial loci encoding gene products that may be advantageous under certain conditions, so called contingency loci (Moxon et al., 1994). In γ-Proteobacteria, Dam methylation regulates not only phase variation and DNA repair, but also bacterial chromosome replication, nucleoid segregation, transposition of insertion elements, and transcription of specific genes (Wion and Casadesús, 2006). Dam MTases are conserved in many pathogenic bacteria, such as *Vibrio cholerae, Salmonella enterica, E. coli, Yersinia pestis*, or *H. influenzae* (Julio et al., 2001). The control of the *dam* gene expression appears to be very tight, and all disorders within the gene expression lead to increased mutation frequency, changes in transposition frequency or to a reduced viability of the mutants. Dam is essential for the viability of *Yersinia pseudotuberculosis* and *V. cholerae* (Julio et al., 2001). The lack of the Dam methylase or its overproduction in *E. coli* results in an increase of spontaneous mutations. Whereas, Dam-deficient mutants of *S. enterica* are non-pathogenic and are effective as live attenuated vaccines for chickens (Dueger et al., 2001).

Intriguingly, pathogenic *N. gonorrhoeae* FA1090 lacks Dam activity. The *dam* gene is inactivated by insertion of the “*dam replacing gene*” (*drg*). The same insertion was observed in many strains of *Neisseria meningitidis* (Jolley et al., 2004). In the *N. meningitidis* B MC58 strain, the *drg* gene encodes a restriction enzyme, NmeBII, which is similar to the *Streptococcus pneumoniae* DpnI restriction endonuclease (Cantalupo et al., 2001). The importance of *drg* locus was recently pointed out in *N. gonorrhoeae* MS11 by Remmele et al. (2014), who describes this region as sensitive to insertional disruption.

In our work we have focused on investigating what could be the advantage of the *drg* gene insertion and its presence within the *dam* gene. We demonstrate that the *ngo0007* gene encodes the Drg endonuclease, exhibiting specificity for GmeATC sequence. Deletion of *drg* gene changes the level of expression of many genes, causes unusual growth, abnormal biofilm production, and generally reduced viability of the gonococcal mutant. Insertion of the *dam* gene, in the *drg* locus, restores growth and viability of the studied *Neisseria* to the level characteristic for the parental strain. Nevertheless, the *dam*-expressing mutant presents reduced adhesion to human epithelial cells *in vitro*.

In conclusion, expression of the *ngo0007* gene encoding the Drg endonuclease is essential for *N. gonorrhoeae* FA1090 growth, biofilm formation and adhesion to human epithelial cells.

## Materials and methods

### Bacterial strains and growth conditions

*E. coli* strain Top10 [F'[lacI^q^ Tn10(Tet^r^)] *mcrA Δ(mrr-hsdRMS-mcrBC) φ80lacZΔM15 ΔlacX74 nupG recA1 araD139 Δ(ara-leu)7697 galE15 galK16 rpsL(Str^r^) endA1 λ^−^*] and GM2163 [*F^−^dam-13::Tn9* (Cam^r^) *dcm-6 hsdR2* (r^−^_k_m^+^_*k*_) *leuB6 hisG4 thi-1 araC14 lacY1 galK2 galT22 xylA5 mtl-1 rpsL136* (Str^r^) fhuA31 tsx-78 glnV44 mcrA mcrB1] were used. *E. coli* strains were grown in Luria-Bertani (LB) broth (Difco) at 37°C with agitation or on LB agar plates, supplemented, when needed, with the following antibiotics: 30 μg/ml kanamycin or 34 μg/ml chloramphenicol or 100 μg/ml ampicillin.

*N. gonorrhoeae* strains were grown on GCB agar base (Difco) supplemented with 1% hemoglobin and 1% *Kellogg's* supplement at 37°C in 5% CO_2_ or in GCB broth supplemented with *Kellogg's* supplement and 0.043% NaHCO_3_ (Dillard, 2006).

### Construction of *N. gonorrhoeae* mutant strains: *drg*-deficient mutant (*drg::cm*) and *dam*-expressing mutant (*drg::dam*)

The mutants were constructed by replacing the wild-type gene on the chromosome with a disrupted allele. The *drg* gene (*ngo0007*) with adjacent region (3237 bp) was amplified by PCR from chromosomal DNA isolated from *N. gonorrhoeae* FA1090 (Gene Bank: AE004969, ATCC 700825) with primers DrgFL (see Supplementary Material, Table 1 for primer sequences), using PfuUltra II Fusion polymerase (Agilent Technologies), according to the manufacturer's specification. The obtained fragment was cloned into the PstI—HindIII site of the pUC19 vector. This construct was transformed into *E. coli* GM2163 cells as *drg* expression proved to be toxic to *dam*^+^ *E. coli* cells. The resulting plasmid was recombined with a chloramphenicol-resistance (*cm*) cassette from the pKRP10 plasmid (Reece and Phillips, 1995) using the BclI site within the *drg* gene. The obtained plasmid was linearized with KpnI and used to transform competent, i.e., piliated *N. gonorrhoeae* cells. The linearization forces the occurrence of double crossing over and replacement of wild-type *drg* gene by *drg::cm* gene. *N. gonorrhoeae* FA1090 *drg*-deficient colonies (*drg::cm* mutants) were selected on GCB plates containing 0.5 μg/ml chloramphenicol.

The *dam* gene (*nmc0327*) with adjacent region (2465 bp) was amplified from *N. meningitidis* FAM18 (Gene Bank: AM421808, ATCC 700532) chromosomal DNA with primers Dam18 (Table S1), using PfuUltra II Fusion polymerase (Agilent Technologies), according to the manufacturer's specification. The obtained fragment was cloned into the SalI—SmaI site of the pUC19 vector. The resulting plasmid was termed pUCDamFam18 and transformed into *E. coli* GM2163 cells as *dam* overexpression was shown to be toxic for *dam*^+^ *E. coli* cells. A kanamycin-resistance cassette from the pDIY-km plasmid (Dziewit et al., 2011) was inserted within the BpiI site of pUCDamFam18 (*dam* gene remained intact). The obtained plasmid was linearized with NdeI and used to transform competent *N. gonorrhoeae* FA1090 *drg::cm* cells. *Dam*-expressing *N. gonorrhoeae* (*drg::dam*) cells were selected on GCB plates containing 30 μg/ml kanamycin. Deletion of the *drg* gene and introduction of the *dam* gene into *N. gonorrhoeae* FA1090 were verified by PCR, sequencing and Southern Blot. With DrgFL primers we performed PCR on genomic DNA isolated from *drg::cm* mutants and in this case we obtained only one PCR product of 4100 bp, corresponding to *drg* gene (3237 bp) interrupted by *cm* cassette (~900 bp).

### Southern blot detection of antibiotic cassettes

Chromosomal DNA was extracted from wild-type (wt) *N. gonorrhoeae* FA1090, *drg::cm* and *drg::dam* mutants. These DNAs (1.0 μg) were digested with MluI, according to the manufacturer's instructions. Restriction fragments were separated on 0.7% agarose gels at 130 V for 2 h. Southern alkali transfer and hybridization analysis were carried out as described by Sambrook and Russell (2001) using non-radioactively labeled (DIG-High Prime, Roche) kanamycin or *cm* cassette. The procedure was carried out according to the manufacturer's recommendations (Roche).

### Field emission scanning electron microscopy

*N. gonorrhoeae drg::cm, drg::dam* and FA1090 wt strains were grown on GCB plates for 24 h. Cells were then harvested and cell suspensions in GCB broth were made to OD_600_ = 0.05 (10^7^ cells/ml). Cell suspensions (5 ml) of each strain were cultivated for 24 h at 37°C in 5% CO_2_ in GCB broth on cover glasses placed in Petri dishes. To fix the formed biofilms, samples were soaked in 3% glutaraldehyde in 0.1 M cacodylate buffer (pH 7.3) for 24 h. Next, biofilms were rinsed five times with pure cacodylate buffer. Duration of the first washing was 60 min, while the next four washings lasted 30 min each. Samples were dehydrated by dipping in 96% ethanol for 6 h, and air-dried. After plasma coating with gold-palladium (circa 2–4 nm thick), biofilms were analyzed with the Field Emission Scanning Electron Microscope (FE SEM) (MERLIN Carl Zeiss Germany) at 2–5 kV range accelerating voltage. The morphology of samples was investigated at various magnification ranges up to nanometer image resolution, using Secondary Electron detectors—conventional side (ET) and In-lens.

### Live cell confocal microscopy

Scanning Confocal Laser Microscopy (SCLM) was used to quantify biofilm development on Glass Bottom Microwell Dishes (35 mm diameter, 20 mm Microwell, no. 1.5 coverglass, 0.16–0.19 mm; MatTek Corporation). *N. gonorrhoeae* strains were grown overnight on GCB agar. Bacterial cells were then flooded with GCB medium with *Kellogg's* supplement and 5 mM MgSO_4_ and transferred to a sterile tube. Bacterial cultures were diluted in GCB broth to OD_600_ = 0.05 and aliquots of these cell suspensions were transferred to dishes and incubated at 37°C in 5% CO_2_ for 16 h. After incubation, the medium was removed and the biofilm that has developed on the bottom of the dishes was washed three times with 10 mM MgSO_4_ and then stained by addition of acridine orange solution (10 μg/ml in 10 mM MgSO_4_). After 30 min of staining, the biofilm was rinsed twice with 10 mM MgSO_4_. Confocal microscopy was conducted using a Nikon Eclipse Ti (A1) microscope equipped with a ×60, 1.4 NA oil immersion phase-contrast lens. An argon laser with a maximum-emission line at 488 nm was used as the excitation source. Horizontal optical thin sections were collected at 0.21 μm intervals from outer surface of the biofilm to the bottom of the glass plate. These images were captured by the *NIS-ELEMENTS* interactive software and three-dimensional reconstructions were created.

### Microtiter-plate adherence assay

Microtiter-plate adherence assay was performed according to Stepanovic et al. with modifications (Stepanovic et al., 2000). *N. gonorrhoeae drg::cm, drg::dam* and FA1090 wt strains were grown overnight on GCB agar. Cells were then harvested and 10^7^ cells/ml suspensions were made in GCB broth with *Kellogg's* supplement and 5 mM MgSO_4_. Bacterial suspensions (0.1 ml) were added into 96-well microtiter plates. Bacterial culture growth was continued for 24 h at 37°C in 5% CO_2_. Then, the content of the wells was aspirated, and each well was washed three times with sterile physiological saline. Plates were vigorously shaken in order to remove all non-adherent bacteria. The remaining attached bacteria were fixed with ethanol for 15 min. After air-drying, adherent bacteria were stained for 10 min with 0.8% crystal violet. Excess stain was rinsed off by placing the plate under running tap water, plates were air dried, and the dye bound to the adherent cells was resolubilized with 0.15 ml of 96% ethanol per well. The crystal violet solubilized by ethanol was transferred to a fresh plate and OD was measured at 570 nm using an automated Sunrise microplate reader and Magellan software (Tecan). All tests were carried out three times and results were averaged.

### RNA isolation

*N. gonorrhoeae drg::cm, drg::dam* and FA1090 wt strains were grown on plates for 24 h as described above. Cells were then harvested from one plate and total RNA was isolated using the High Pure RNA Isolation Kit (Roche), according to the manufacturer's recommendations. Contaminating DNA were removed using DNA-*free™*, DNase Treatment & Removal (Ambion), according to the manufacturer's instructions. RNA concentration and RNA quality were measured using NanoDrop 2000 (Thermo Scientific) and 2100 Bioanalyzer (Agilent Technologies).

### Microarray experiments

DNA microarray analysis was performed using an Agilent custom-designated 60-mer oligonucleotide array (Agilent-034141). *N. gonorrhoeae* microarrays were designed as 8000 × 15,000 oligonucleotide chips, which included all ORFs from the genome of the *N. gonorrhoeae* FA1090 strain (accession number AE004969). Probe design was performed taking into account the oligonucleotide sequence specificity and structural and thermodynamic constraints.

Two-color microarray was used to examine transcriptional profiles of the studied gonococcal strains. The Low Input Quick Amp Labeling WT (LIQA WT) Kit (Agilent Technologies) was used to amplify and label whole transcripts. Additionally, the RNA Spike In Kit for Two color v4.0 (Agilent Technologies) that provides positive controls for monitoring the microarray workflow from sample amplification and labeling to microarray processing was used as internal control. The degree of hybridization between the spike-ins and the control probes was used to normalize the hybridization measurements for the target probes. The procedure using the LIQA WT kit was performed according to manufacturer's instructions. Briefly, the method involved the following steps: (i) cDNA synthesis using total RNA (50 ng) as template; (ii) cRNA synthesis and labeling using cDNA as template, T7 RNA polymerase, NTPs and fluorescent dye. Simultaneously two mixtures were prepared, one reaction with Cy3 as fluorescent dye, the second one with Cy5; (iii) purification of labeled cRNA and measurement of the efficiency of incorporation of the Cy5 or Cy3 dye. The Cy3 dye was used to label the cRNA wt strain, and the Cy5 dye to label the cRNA *N. gonorrhoeae* mutant strain. RNeasy Mini Kit (Qiagen) was used for purification of the amplified cRNA samples. Quantity of cRNA was determined by spectrophotometry using NanoDrop. Next, equal amounts of Cy5- and Cy3-labeled cRNAs were hybridized onto the microarray for 17 h at 65°C and hybridized microarrays were washed according to the Agilent protocol. Gene Expression Hybridization Kit and Gene Expression Wash Buffer Kit were used respectively to hybridize and wash the hybridized microarray. Microarray slides were scanned using the Agilent G2565CA Microarray Scanner System.

### Microarray analysis

Data files generated by the Agilent G2565CA Microarray Scanner System (Agilent Technologies) were imported into the GeneSpring version 12.5 (Agilent Technologies). Normalized data from all samples were filtered on genes flagged as present or marginal with the resulting gene list used for further gene expression analysis and clustering (Student's *t*-test against zero, asymptotic *P*-value computation and Benjamini Hochberg FDR multiple testing correction). Changes were expressed as the ratio of gene expression of wt FA1090 strain over expression of the same gene for the mutant. Genes with differential expression of ≥1.5-fold and a *P*-value < 0.05 were selected. Supporting microarray data for all studied gonococcal mutant strains have been deposited in the NCBI's Gene Expression Omnibus (Edgar et al., 2002), and are accessible through GEO series accession number GSE60347 (www.ncbi.nlm.nih.gov/geo/query/acc.cgi?acc=GSE60347).

### Real-time qRT-PCR

cDNAs were obtained by reverse transcription of 10 μg of total RNA using the Maxima First Strand cDNA Synthesis Kit for qPCR-RT, according to the manufacturer's instruction (Thermo Scientific). Real-time PCR using 5×HOT FIREPol® EvaGreen® qPCR Mix Plus (ROX) (Solis BioDyne) was carried out on The Applied Biosystems® StepOne™ Real-Time PCR Systems (Life Technologies). HPLC purified oligonucleotide primer pairs specific for each gene of interest (Supplementary Material, Table 1) were purchased from Sigma-Aldrich. Relative quantification of gene transcription was performed using the comparative Ct (threshold cycle) method.

The relative amount of target cDNA was normalized using the 16S rRNA gene as an internal reference standard and primers 16S (Supplementary Material, Table 1). Data presented are averages of at least four independent repetitions.

### Gonococcal cell viability assay

*N. gonorrhoeae drg::cm, drg::dam* and FA1090 wt strains were grown overnight on GCB agar with *Kellogg*'s supplement at 37°C in 5% CO_2_. Cells were then harvested from plates and cell suspensions in GCB broth with 5 mM MgSO_4_ were made (10^7^ cells/ml). Bacterial culture growth was continued at 37°C and with 50 rpm shaking. Samples were taken every hour for the first 7 h. The number of viable bacterial cells in the cultures was determined by quantitative determination of ATP present in the cells using the BacTiter-Glo™ Microbial Cell Viability Assay (Promega), according to the manufacturer's protocol. Analysis of the amount of ATP was carried out on the basis of ATP oxidation reaction carried out by luciferase. Luciferase in the presence of oxygen and ATP causes emission of photons, which is proportional to the amount of ATP molecules. Signal detection was performed using the Promega luminometer (Glomax multi+ with luminescence module). All tests were carried out three times and results were averaged.

### Adhesion assay of *N. gonorrhoeae* to human cells

The human adenocarcinoma endometrial cell line Hec-1-B (ATCC HTB113) was purchased from the American Type Culture Collection and maintained in DMEM medium (Sigma-Aldrich) containing 2 mM glutamine (Cytogen) supplemented with 10% fetal bovine serum (Cytogen) and 1 mM sodium pyruvate (Sigma-Aldrich) at 37°C in 5% CO_2_. Cell lines were passaged every 5 days.

Hec-1-B cells were seeded in 24-well plates (Falcon) at a density of 10^5^ cells/well and incubated for 3 days at 37°C in 5% CO_2_. Bacteria were harvested from GCB agar plates and suspended in GCB broth. Such suspension was used to infect the human cells at a multiplicity of infection of 10. The medium was discarded from one set of wells at 3.5 h postinfection, and cells were washed six times with pre-warmed PBS. Human cells were lysed with GCB medium containing 0.5% saponin (Sigma-Aldrich), and dilutions of the lysates were plated on GCB agar for enumeration of human cell-associated colony forming units (CFU). At the same time, the medium was removed from a parallel set of infected cultures, cells were diluted and plated on GCB agar for enumeration of CFU. Cultures from this parallel set of wells were also lysed with saponin and dilutions plated on agar for CFU enumeration. The sum of CFU from the medium supernatant and from the cell lysates accounted for the total CFU. The adhesion index was calculated by dividing the number of cell-associated CFU by the number of total CFU (Hopper et al., 2000). All tests were carried out three times and results were averaged.

### Cloning, expression and *in vitro* characterization of the *drg* endonuclease

Cloning of the *drg* gene was performed in *E. coli* GM2163 cells. The *ngo0007* gene (spanning from 9365 to 10,294 nt in the *N. gonorrhoeae* FA1090 genome) was amplified by PCR from chromosomal DNA of *N. gonorrhoeae* FA1090 and cloned into the NheI—HindIII sites of pET28a (Novagen), resulting in formation of the pET28a::*drg* plasmid. In this construct, the expressed Drg endonuclease contained a His tag on the amino terminus. PCR reactions were carried out using DrgpET primers (Supplementary Material, Table 1) and Pfu DNA polymerase (Thermo Scientific), according to the manufacturer's recommendations. To construct the *dam^−^ E. coli* strain suitable for expression of the *drg* gene, *E. coli* GM2163 cells and λ DE3 Lysogenization Kit (Novagen) were used according to the manufacturer's protocol. The λ DE3 Lysogenization Kit is designed to construct *E. coli* strains where the gene for T7 polymerase is localized on the chromosome under the *lacUV5* control.

To express and purify the Drg endonuclease, a single colony generated by fresh transformation of *E. coli* GM2163 (DE3) (pET28a::*drg*) was used to start an overnight culture at 37°C in LB broth with kanamycin and 1% glucose. Then, the bacterial culture was refreshed to OD_600_ = 0.05 in LB with kanamycin and incubated at 37°C. When the OD_600_ of the culture reached 0.6, IPTG was added to a final concentration of 1 mM. Incubation was continued at 18°C for an additional 3 h. Cells were collected by centrifugation and the bacterial pellet was resuspended in 10 ml of buffer containing 50 mM NaHPO_4_, 500 mM NaCl, 20 mM imidazole, 10 mM β-mercaptoethanol, 0.1% Tween 20, 55 μM PMSF and 1 tablet of complete Mini, EDTA-free (protease inhibitor cocktail) (Roche). After sonication, the cellular debris was removed by centrifugation at 40,000 *g* for 1 h and the supernatant applied to a Ni-NTA Agarose column previously equilibrated with the above buffer. The column was washed with buffer containing 50 mM NaHPO_4_, 500 mM NaCl, 35 mM imidazole and 10% glycerol. Proteins were eluted with a gradient of imidazole (50 mM–0.5 M) in the same buffer. The Drg protein was eluted at 0.2–0.25 M imidazole. The pre-purified enzyme was resolved by electrophoresis on a 12% SDS PAGE gel. The amount of purified protein was determined using the Bradford Reagent (Sigma-Aldrich) with bovine serum albumin (BSA) as a protein standard. PageRuler™ Prestained Protein Ladder (170, 130, 100, 70, 55, 40, 35, 25, 15, and 10 kDa) (Thermo Scientific) was used as a protein molecular weight marker.

Endonuclease activity was assayed by incubating 0.085 pmol of pUC19 DNA with 2.6 pmol of the enzyme in a final volume of 20 μl containing: 66 mM Tris-acetate (pH 7.9 at 37°C), 20 mM magnesium acetate, 132 mM potassium acetate and 0.2 mg/ml BSA for 60 min at 37°C. The cleaved products were analyzed using 0.7% agarose gels. DNA was visualized by ethidium bromide staining.

### Enzymes, oligonucleotides, and chemicals

Restriction enzymes, T4 DNA ligase, IPTG and DNA and protein markers were purchased from Thermo Scientific and used under conditions recommended by the manufacturer. Kits for DNA purification and plasmid DNA extraction were purchased from A&A Biotechnology, Gdansk, Poland. Ni-NTA Agarose was purchased from Qiagen. All other chemicals were purchased from Sigma-Aldrich, unless otherwise noted. Primers for DNA amplification were obtained from the Institute of Biochemistry and Biophysics (Poland).

### Other methods

All other routine methods were carried out in accordance with protocols described by Sambrook and Russell (2001).

### Computer analysis

DNA and protein sequences were compared with GenBank on the BLAST server hosted by the National Center for Biotechnology Information (www.ncbi.nlm.nih.gov/blast), KEGG and Uniprot databases. Clusters of Orthologous Groups (COG) were assigned with the NCBI Conserved domains server (http://www.ncbi.nlm.nih.gov/Structure/cdd/wrpsb.cgi).

## Results

### Localization of the *drg* gene in *neisseriaceae* genomes is associated with the *leuS* locus

**Figure 1:**
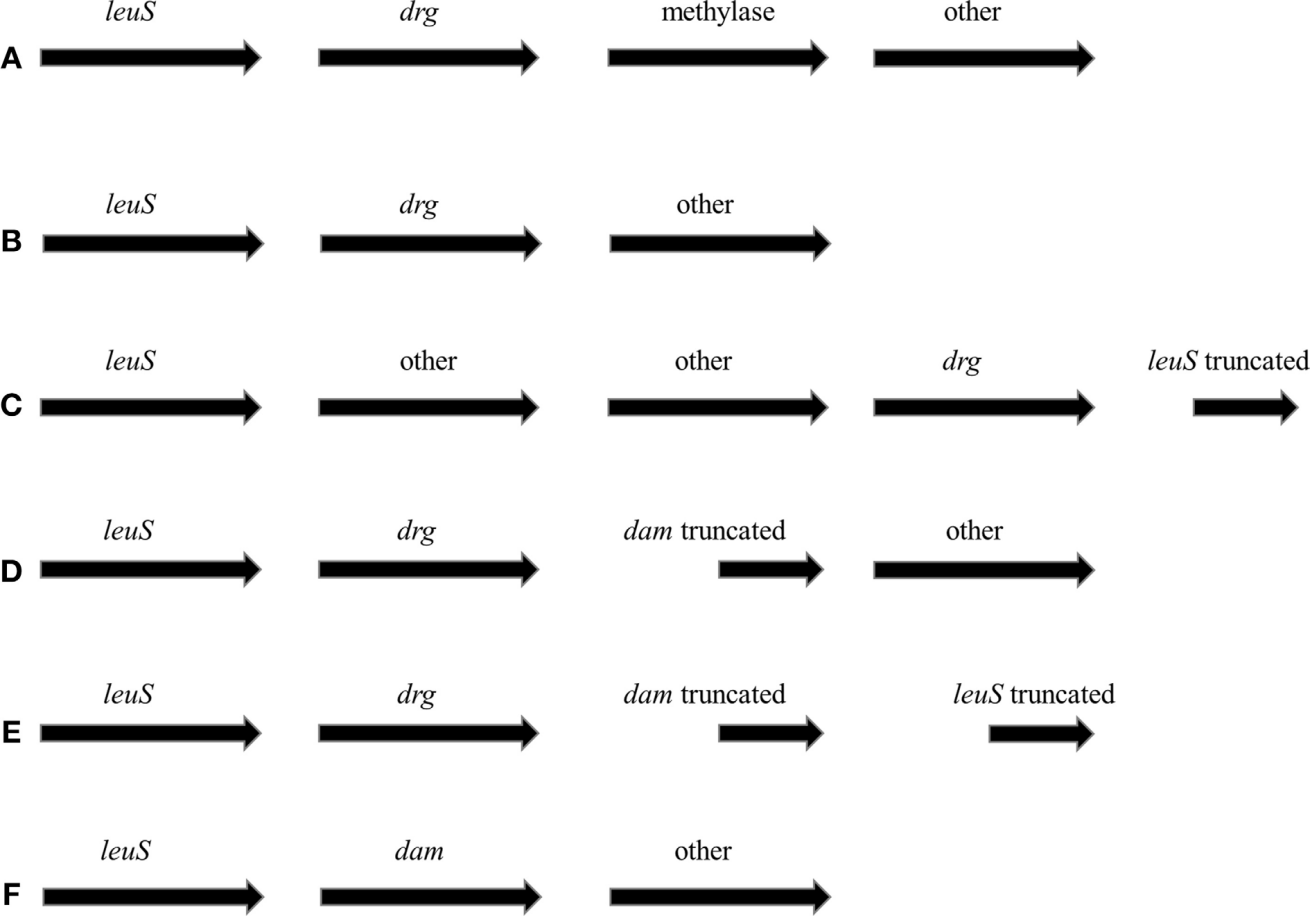
**Genetic maps of *drg* or *dam* loci in the genomes of *Neisseriaceae***. Analysis included only the wholly-sequenced genomes of *Neisseriaceae*. *leuS*–gene encoding leucyl-tRNA synthase, *drg*—*dam replacing gene, dam*—gene encoding Dam methylase. For each type of locus organization, **(A–F)**—species and serogroup names of *Neisseriaceae* are given in the text.

The *drg* gene was previously identified in the genomes of *N. meningitidis* and *Neisseria lactamica* (Cantalupo et al., 2001; Jolley et al., 2004). In *N. gonorrhoeae* FA1090, the Drg endonuclease seems to be encoded by the *ngo0007* gene. By blasting the nucleotide sequence of the *drg* locus against the NCBI database 27 hits were obtained. Among them, 23 ORFs were *drg*. Within *Neisseriaceae*, only *N. gonorrhoeae, N. meningitidis*, and *N. lactamica* were determined to contain the *drg* locus. Only 21 hits were found in wholly-sequenced neisserial genomes. For these genomes, we have analyzed the genetic context of the *drg* and *dam* loci. In all cases, the *drg*/*dam* locus neighbored the *leuS* locus (leucyl-tRNA synthetase) (Figure 1). In three cases (*N. gonorrhoeae* FA1090, MS11, and TCDC-NG08107), the *drg* gene was followed by a type II DPNIIB—like enzyme (Figure 1A). Such organization is reminiscent of minimal mobile elements, where an acquired cassette-like is located between highly conserved flanking genes (Saunders and Snyder, 2002). The majority of these cassettes encode restriction and modification systems (Snyder et al., 2007). However, no small repeated elements with some transposon-like properties were found in proximity of the *ngo0007* locus (Correia et al., 1988). The same organization was present in *N. meningitidis* NCCP11945, M01-240149, M01-240196, H44/76, NZ-05/33, 510612, WUE2594, and 8013 strains, but the *drg* gene was followed by different hypothetical genes (Figure 1B). In case of *N. lactamica* 020-06 and *N. meningitidis* alpha 522, the *drg* gene with other genes was flanked by *leuS* on both sides (Figure 1C). For *N. meningitidis* MC58, 053442, Z2491, and alpha710, the *drg* gene was inserted within the *dam* locus (Figures 1D,E), resulting in *dam* truncation. In the genomes of *N. meningitidis* strains MC58, 053442, and alpha710, the truncated *dam* gene was followed by a truncated *leuS* gene, suggesting that *dam* is also an acquired locus in these bacteria. For four strains, *N. meningitidis* G2136, FAM18, alpha14 and M01-240355, the *drg* locus is absent and *leuS* is followed by an intact *dam* gene (Figure 1F).

### Construction of *N. gonorrhoeae drg*-deficient and *dam*-expressing mutants

As mentioned previously, in most *Neisseriaceae* the *drg* gene is inserted in the *dam* locus. Dam MTase activity excludes the coexistence of the Drg endonuclease activity. As we decided to study gene expression and some physiological aspects in the presence of Dam activity compared to the wt *N. gonorrhoeae* strain, two gonococcal mutants were constructed. Mutants were obtained by the gene replacement method using suicide plasmids. A plasmid with the *drg* gene interrupted by the *cm* cassette was used for inactivation of the *drg* gene, which resulted in the *N. gonorrhoeae drg::cm* strain. To obtain the *N. gonorrhoeae drg::dam* mutant, a plasmid with the cloned *dam* gene was used. PCR, sequencing and Southern Blot were used to verify whether the constructed mutant strains contain only the desired mutations (data not shown). Additionally, we confirmed the activity of Dam MTase, introduced by insertion of the *dam* gene, by extracting the total genomic DNA from the *drg::dam* mutant and subjecting it to digestion with enzymes that are sensitive to GmeATC methylation. Genomic DNA isolated from the *N. gonorrhoeae drg::dam* mutant was digested by DpnI (cuts only GmeATC), but not by MboI (cuts only unmethylated GATC). In contrast, analysis of the *N. gonorrhoeae* FA1090 wt genomic DNA, digested by restriction enzymes sensitive to GmeATC (DpnI or MboI) showed that it is not methylated at GATC sites (data not shown).

### Presence of the *drg* gene influences *N. gonorrhoeae* viability

**Figure 2:**
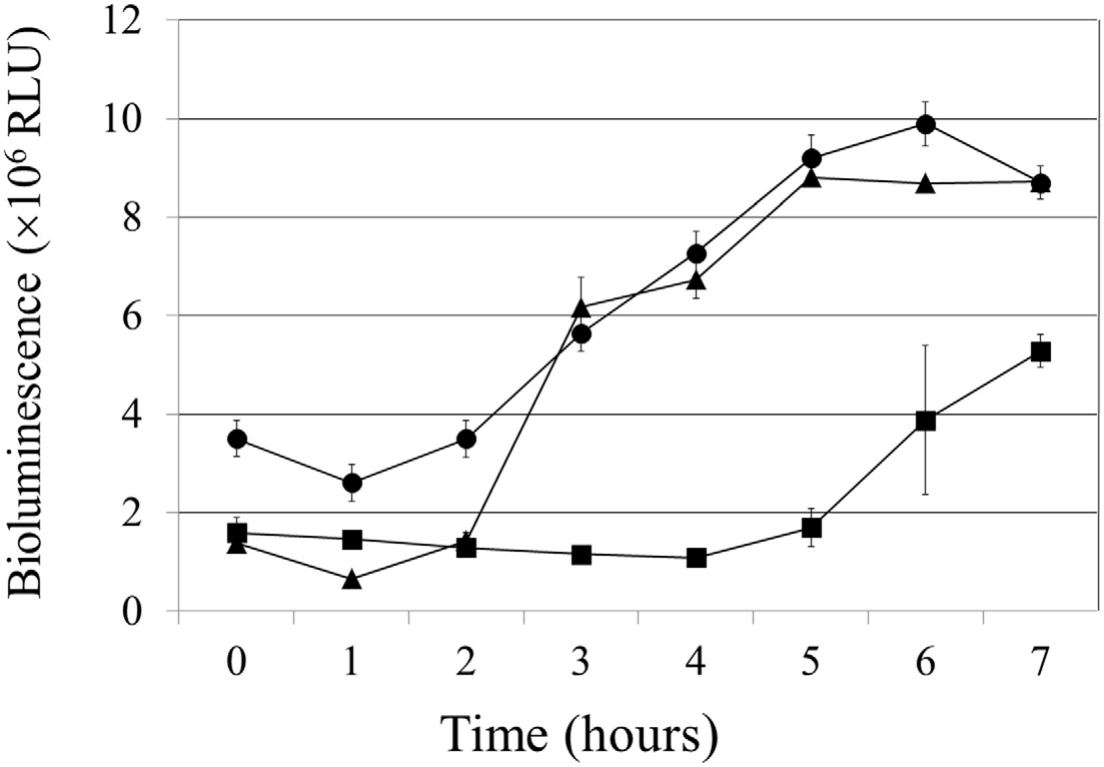
**Effect of *drg* disruption or *dam* insertion on *N. gonorrhoeae* growth**. Growth and viability of wild-type *N. gonorrhoeae* (triangle), *drg::cm* mutant (square), and *drg::dam* mutant (circle) were measured for 7 h using the BacTiter-Glo™ Microbial Cell Viability Assay kit. Bioluminescence was directly proportional to the amount of viable cells.

To measure the viability of *N. gonorrhoeae*, the BacTiter-Glo™ Microbial Cell Viability Assay was used. In the assay, due to the presence of ATP in living cells, a luminescent signal, proportional to the number of viable cells in the culture, is generated. *N. gonorrhoeae* cells were grown in liquid medium with gentle agitation. After 2 h of lag phase, the wt gonococcal cells kept growing exponentially until a plateau phase was achieved after approximately 5 h (Figure 2). Growth of the *N. gonorrhoeae drg::dam* mutant was very similar to the growth of the wt strain.

Inactivation of the *drg* gene significantly decreased the *N. gonorrhoeae* growth rate, *drg::cm* cells remained in the lag phase for 5 h. While, absence of the *drg* gene in the presence of the *dam* gene seemed have no effect on bacterial growth as compared to wt cells.

### Impact of *drg* and *dam* gene on gonococcal gene expression

**Figure 3:**
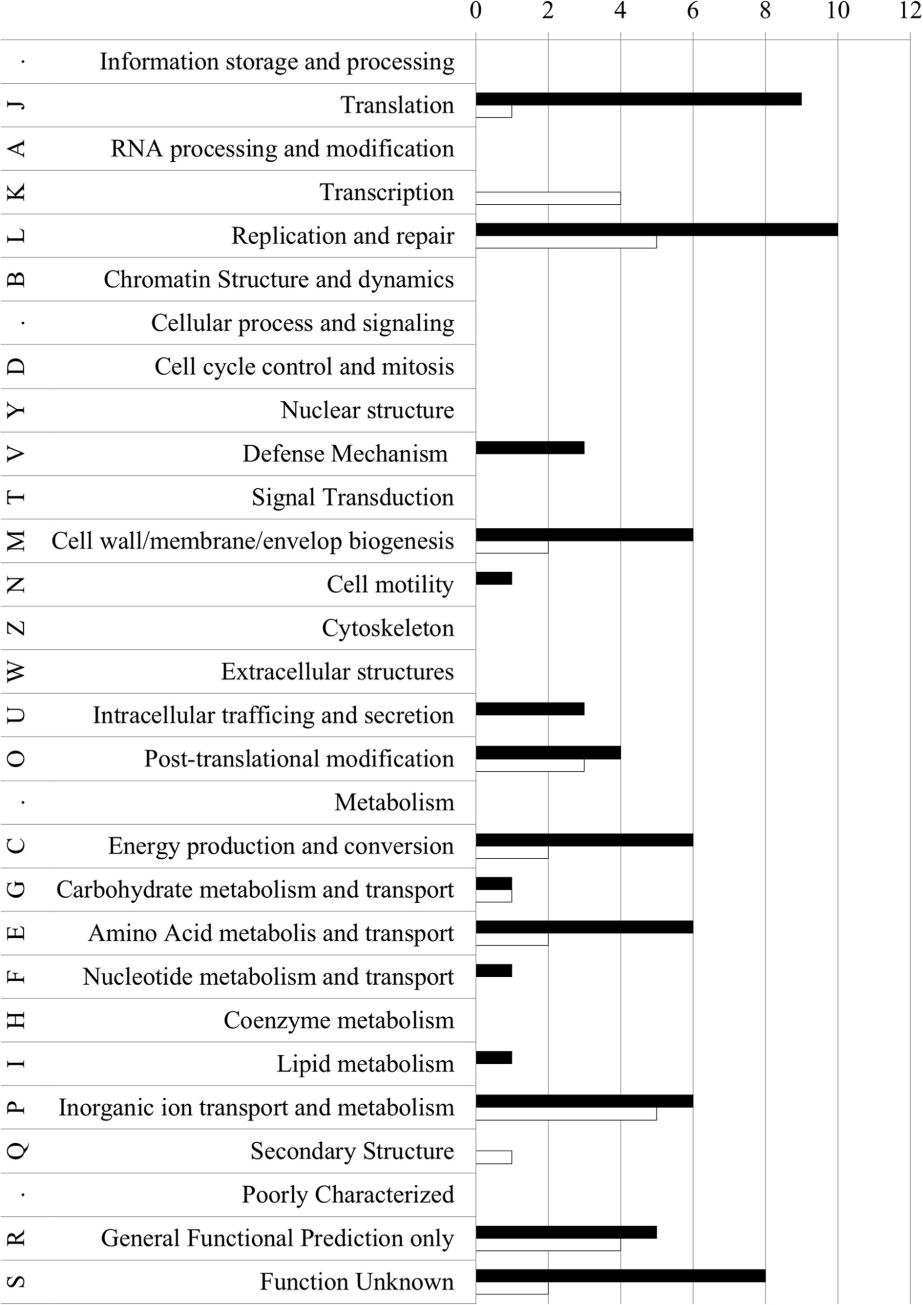
**COGs classification of proteins encoded by up- and down-regulated genes in the *drg*-deficient *N. gonorrhoeae* vs. wild-type strain**. Letters represent COGs categories; numbers—the number of proteins in each category; black bars–proteins encoded by up-regulated genes; white bars–proteins encoded by down-regulated genes.

The gonococcal gene expression was determined by expression microarray analysis. For the *N. gonorrhoeae drg::cm* mutant, a total of 195 genes exhibited an altered expression by at least 1.5 fold compared to the wt strain (Supplementary Material, Table 2). This group of genes represented 8.94% of the total gene pool. Expression of 136 genes (69.74%) was up-regulated and 59 genes (30.26%) were down-regulated. We have determined the cluster of orthologous gene, COG, (Natale et al., 2000; Tatusov et al., 2000) category for each gene (with deregulated expression), which encode proteins of known function (Supplementary Material, Table 2 and Figure 3). We found 102 COGs (70 among up-regulated genes and 32 among down-regulated), while 97 proteins did not exhibit any known conserved domain. As seen in Figure 3, genes which expression was enhanced in the gonococcal *drg::cm* mutant encoded proteins that mostly belong to the “Information storage and processing” or “Metabolism” category. In the first group, 25 proteins belonged to COGs involved in “Replication, recombination and repair” (L category) and “Translation, ribosomal structure and biogenesis” (J category). A total of 27 proteins was grouped in the “Metabolism” category. Among them, we found COGs classified to “Energy production and conversion” (C category), “Amino Acid metabolism and transport” (E category) and “Inorganic ion transport and metabolism” (P category). A predicted general function (R category) was determined for five proteins and eight were classified to the S category (Function Unknown). Detailed information can be found in Supplementary Material, Table 2.

**Figure 4:**
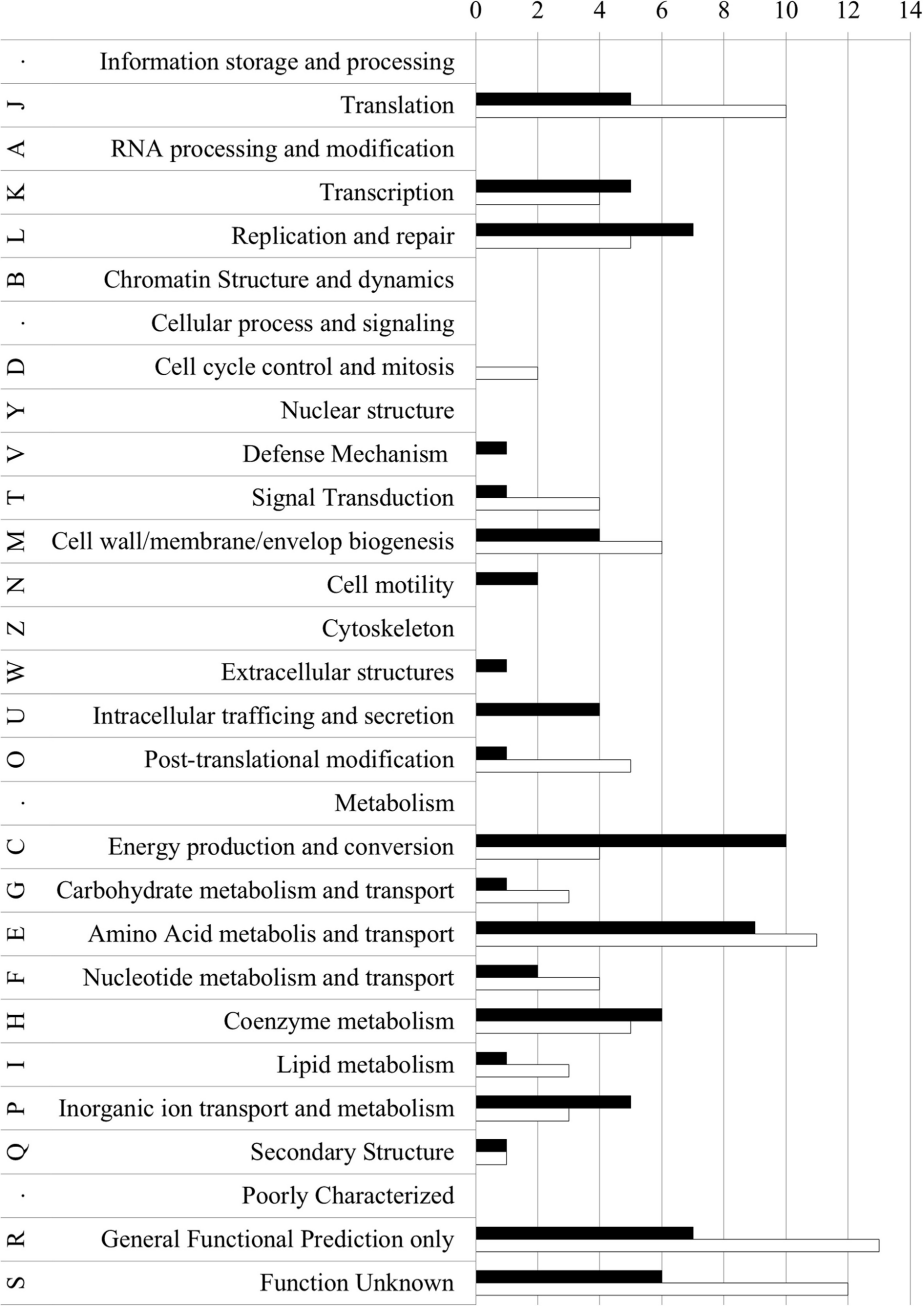
**COGs classification of proteins encoded by up- and down-regulated genes in *N. gonorrhoeae* with inserted *dam* gene vs. wild-type strain**. Letters represent COGs categories; numbers—the number of proteins in each category; black bars–proteins encoded by up-regulated genes; white bars–proteins encoded by down-regulated genes.

Expression microarray analysis was also performed for the *N. gonorrhoeae drg::dam* mutant. Obtained results showed that expression of 240 genes (11% of total genes) was deregulated by at least 1.5 fold (Supplementary Material, Table 3). Expression of 117 genes (48.75%) was increased and expression of 123 genes (51.25%) was decreased in comparison to the wt *N. gonorrhoeae* strain. We have identified 173 functional COGs (Supplementary Material, Table 3 and Figure 4). Most of the proteins which genes were deregulated (9 up- and 11 down-regulated) belonged to the “Amino acid transport and metabolism” (E category). The second most represented category was “Translation, ribosomal structure and biogenesis” (J category) within the “Information storage and processing” group (15 proteins). 21 proteins which genes were deregulated were classified to COGs involved in “Replication, recombination and repair” (L category) and “Transcription” (K category). 14 deregulated genes encoded proteins that matched COGs engaged in “Energy production and conversion”(C category). All identified categories are represented on Figure 4 and in Supplementary Material, Table 3. Many proteins, encoded by deregulated genes, did not exhibit any conserved domain (73 proteins) or their conserved domains did not have a characterized function (38 proteins in R and S category).

Moreover, expression of the 43 same genes was deregulated on the same way in both gonococcal (*drg::cm* and *drg::dam*) mutants. Two genes were regulated differently: *ngo1068*, encoding a Maf-B like protein (up-regulated in *N. gonorrhoeae drg::dam* and down-regulated in *drg::cm* strain) and the *ngo18781* gene, encoding a hypothetical protein (down-regulated in *drg::dam* and up-regulated in *drg::cm*).

### Adherence and biofilm formation by *N. gonorrhoeae drg*-deficient, *dam*-expressing and wt strains

**Figure 5:**
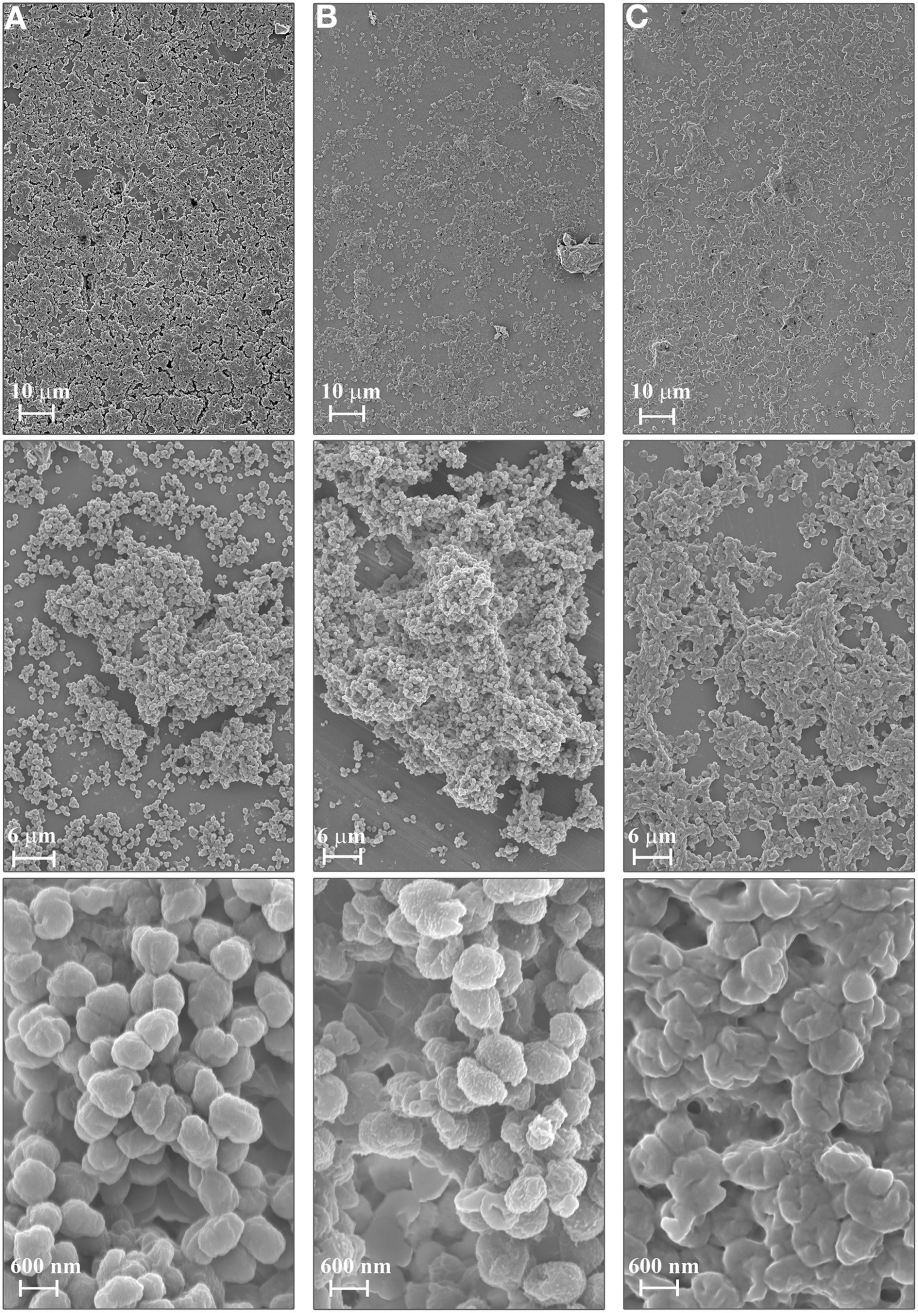
**Biofilm formed by *N. gonorrhoeae* on cover glass after 24 h visualized by Field Emission Scanning Electron Microscopy**. **(A)** Biofilm produced by wild-type FA1090 strain; **(B)** Biofilm produced by *N. gonorrhoeae drg::cm* mutant and **(C)** Biofilm produced by *N. gonorrhoeae drg::dam* mutant. Experiments were triplicated and representative images are shown.

To evaluate the ability of *N. gonorrhoeae* FA1090 wt, *drg::cm* and *drg::dam* mutants to form biofilm, we used FE SEM and SCLM. For FE SEM, gonococci were grown on a cover slip glass for 24 h in standard growth conditions. The wt strain formed a homogenous biofilm covering almost the whole glass with cells associated in layers (Figure 5A). Biofilm presented a structural roughness, yet, cells seems to be attached to each other in a relaxed, dispersed way. Gonococcal cells exhibited on their surface characteristic, discrete vesicles (blebbings) and had proper coffee-bean shape. In contrast, the biofilm formed by the *N. gonorrhoeae drg*-deficient mutant was leakier. Many single cells could be observed with numerous empty spaces between them (Figure 5B). However, some structural clumps were also noted. Such formations were well-structured like in the biofilm produced by wt strain. Cells were covered with many small tabs, and their surface was wrinkled. Biofilm created by the gonococcal *drg::dam* mutant seemed not to adhere to the glass surface as well as the wt strain (Figure 5C). Biofilm formations were “stressed” and seemed to be condensed. Cells were embedded in an extracellular structure that gave a cemented appearance. Single cells were crumpled.

**Figure 6:**
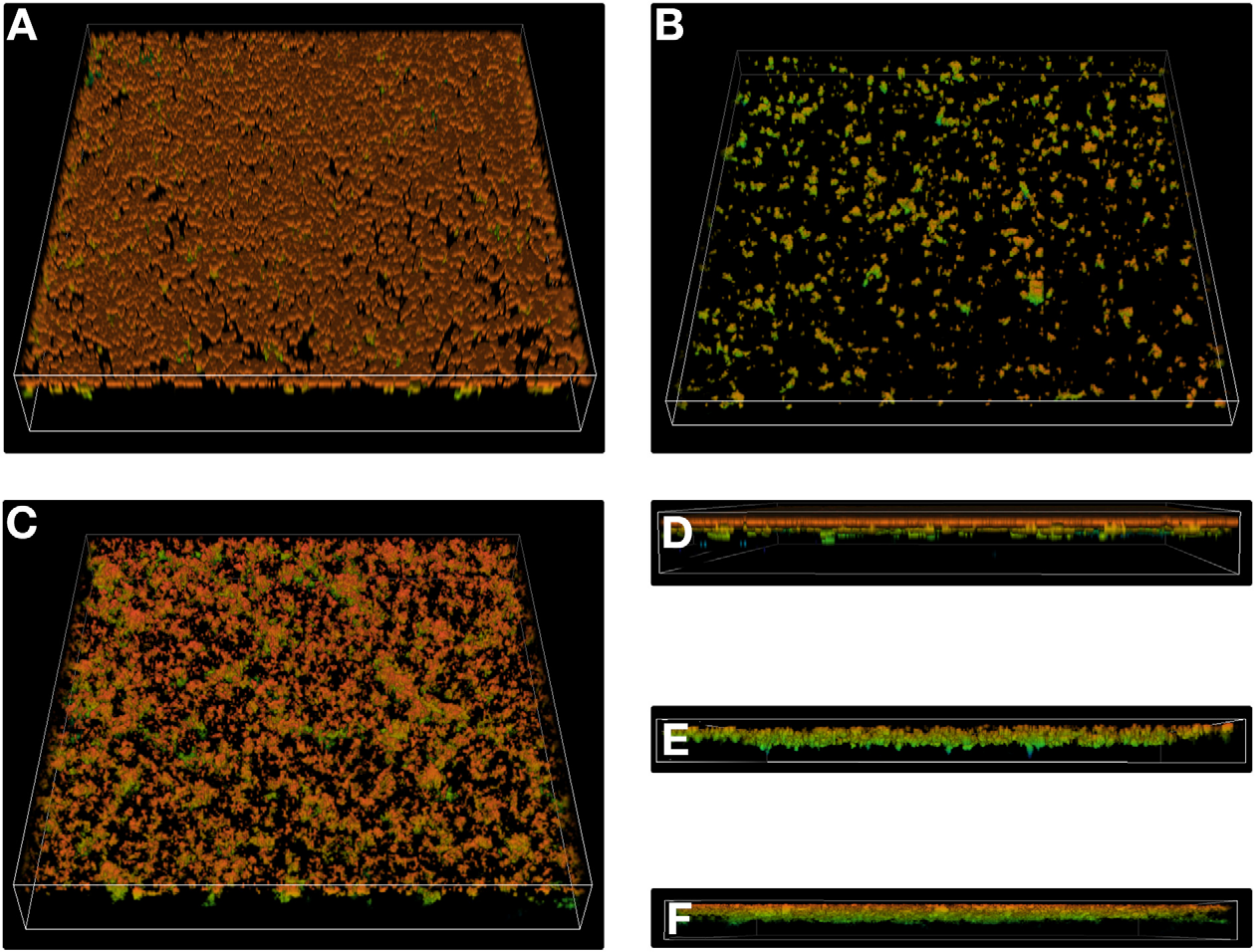
**Three-dimensional structures of biofilms formed by *N. gonorrhoeae* after 24 h visualized by Scanning Confocal Laser Microscopy**. **(A–C)** Three-dimensional biofilm structures and density; **(D–F)** Biofilm thickness. **(A,D)** Biofilm produced by the wild-type strain; **(B,E)** Biofilm produced by the gonococcal *drg::cm* mutant; **(C,F)** Biofilm produced by the gonococcal *drg::dam* mutant. Experiments were triplicated and representative images are shown.

To observe live gonococcal biofilm, we used SCLM. For this purpose, *N. gonorrhoeae* was cultivated on glass plates and after a 24-h growth, cells were stained by acridine orange. As shown on Figure 6, the biofilm formed by the *N. gonorrhoeae drg::cm* mutant was seriously confined (Figure 6B) in comparison to that formed by the wt strain (Figure 6A). The biofilm produced by the wt strain was dense and uniformly distributed on the glass surface. In contrast, the biofilm produced by the gonococcal *drg::cm* strain was represented by scattered clusters. The cross section (Figures 6D,E) indicated that only a small number of cells was attached to the glass. Finally, the biofilm produced by the *N. gonorrhoeae drg::dam* mutant (Figure 6C) resembled the one formed by the wt strain, however, it was less dense and unoccupied spaces between clumps could be noticed. Moreover, the biofilm seemed to be slightly thicker in comparison to the wt biofilm, indicating more complex structures.

**Figure 7:**
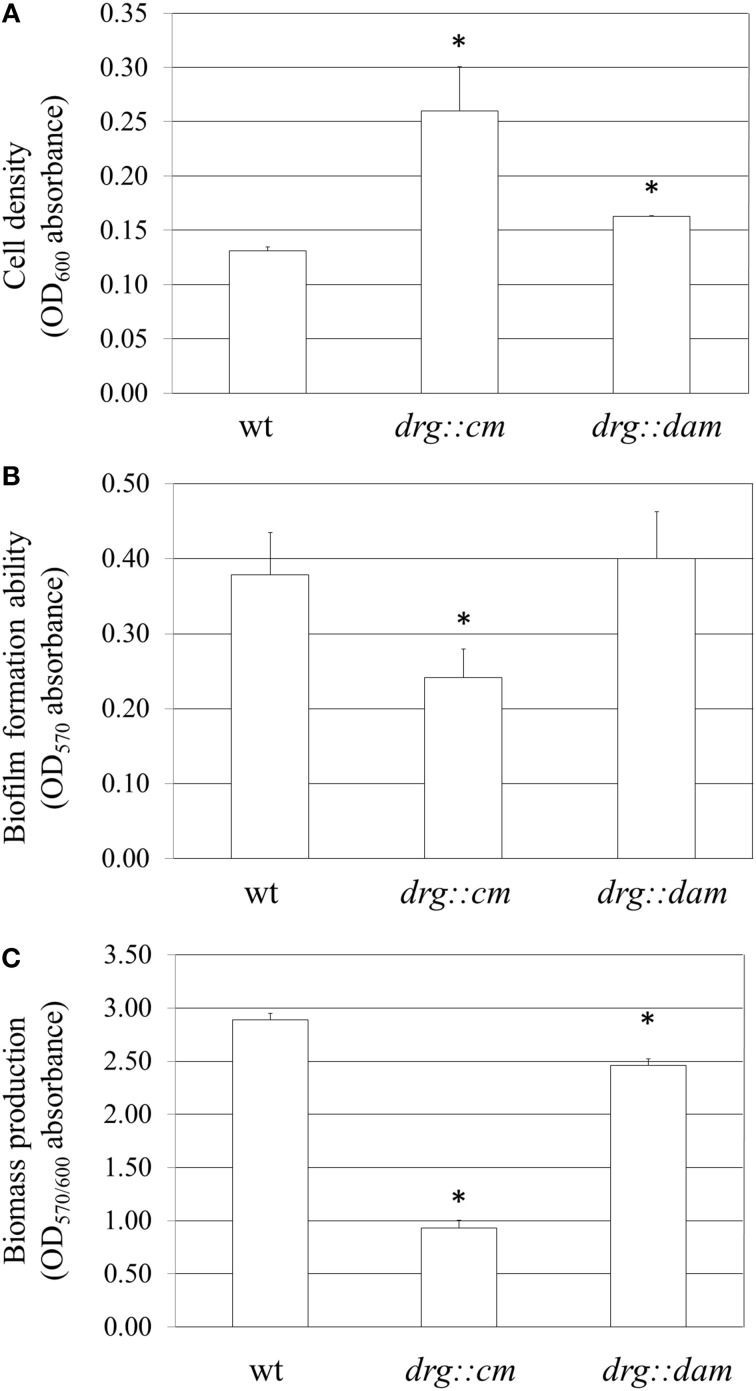
**Growth and biofilm biomass of *drg*-deficient and *dam*-expressing *N. gonorrhoeae* mutants**. Measurements were performed after a 24-h growth without shaking and assayed by crystal violet staining. **(A)** Biofilm and planktonic cell growth was measured at absorbance OD_600_. **(B)** Biofilm biomass was quantitatively measured at OD_570_, after removing planktonic cells. **(C)** Ratio of cells that forms biofilm vs. total grown cells (OD_570/600_). The asterisks represent statistical differences in comparison to the wild-type strain (*P* < 0.05).

Additionally, we measured the growth and adherence of gonococci to polystyrene surfaces. After a 24-h growth, the number of *N. gonorrhoeae drg::cm* cells (Figure 7A) exceeded two fold (OD_600_ = 0.260 ± 0.04) the number of gonococcus wt cells (OD_600_ = 0.131 ± 0.003; *P* < 0.05). The number of *N. gonorrhoeae drg::dam* cells was also increased (OD_600_ = 0.163 ± 0.001) compared to the wt, but the difference was weaker, yet, statistically significant (*P* < 0.05). The amount of bacterial cells that adhered to the examined surface (Figure 7B) was similar for *N. gonorrhoeae drg::dam* (OD_570_ = 0.400 ± 0.062) and wt (OD_570_ = 0.378 ± 0.057) strains, but significantly lower for *N. gonorrhoeae drg::cm* (OD_570_ = 0.242 ± 0.038) in comparison to wt cells (*P* < 0.05).

Both mutants (*drg::cm* and *drg::dam*) formed smaller biofilm biomasses (OD_570/600_ = 0.93 ± 0.078 and 2.46 ± 0.061, respectively; *P* < 0.05) per cell than the wt strain (OD_570/600_ = 2.89 ± 0.059) as determined by comparing the biofilm biomass to total cells (planktonic and forming biofilm) (Figure 7C).

### Adhesion of *N. gonorrhoeae* to human epithelial cells Hec-1-B

**Figure 8:**
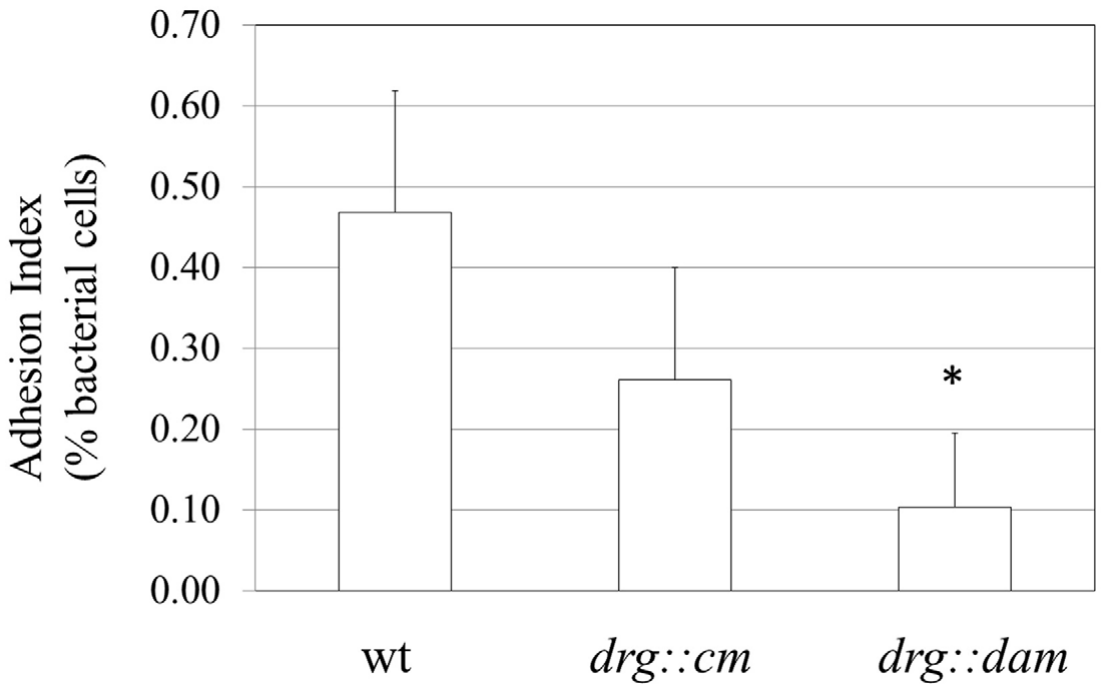
***N. gonorrhoeae* adhesion to human Hec-1-B cells**. Human epithelial cells were infected for 3.5 h with the gonococcal wild-type strain, *drg::cm* or *drg::dam* mutant. The adhesion index was calculated as a ratio between the number of bacteria that adhered to human cells to the total cell number used for infection. The asterisk represents statistical difference in comparison to the wild-type strain (*P* < 0.05).

After demonstrating that gonococcal *drg*-deficient and *dam*-expressing mutant strains differ from the parental strain in adherence to different surfaces (glass and polystyrene), we investigated their ability to attach to human epithelial cells. Hec-1-B cells were cultured to monolayer and infected with *N. gonorrhoeae* for 3.5 h. After incubation, the bacteria that were attached to the human cell membrane or have penetrated into the human cells were liberated. In parallel, we counted all bacterial cells added to the eukaryotic cells (total CFU). Under our experimental conditions, the adhesion index of the *drg::cm* mutant was 0.26 (±0.13) and for the wt strain the adhesion index was 0.47 (±0.30) (Figure 8). However, this difference was not statistically significant. The gonococcal *drg::dam* mutant presented almost a 5-time lower adhesion compared to the wt strain (Figure 8). The adhesion index determined for *drg::dam* strain was 0.102 (±0.09; *P* < 0.05).

### *In vitro* characterization of the *N. gonorrhoeae* FA1090 *drg* endonuclease

**Figure 9:**
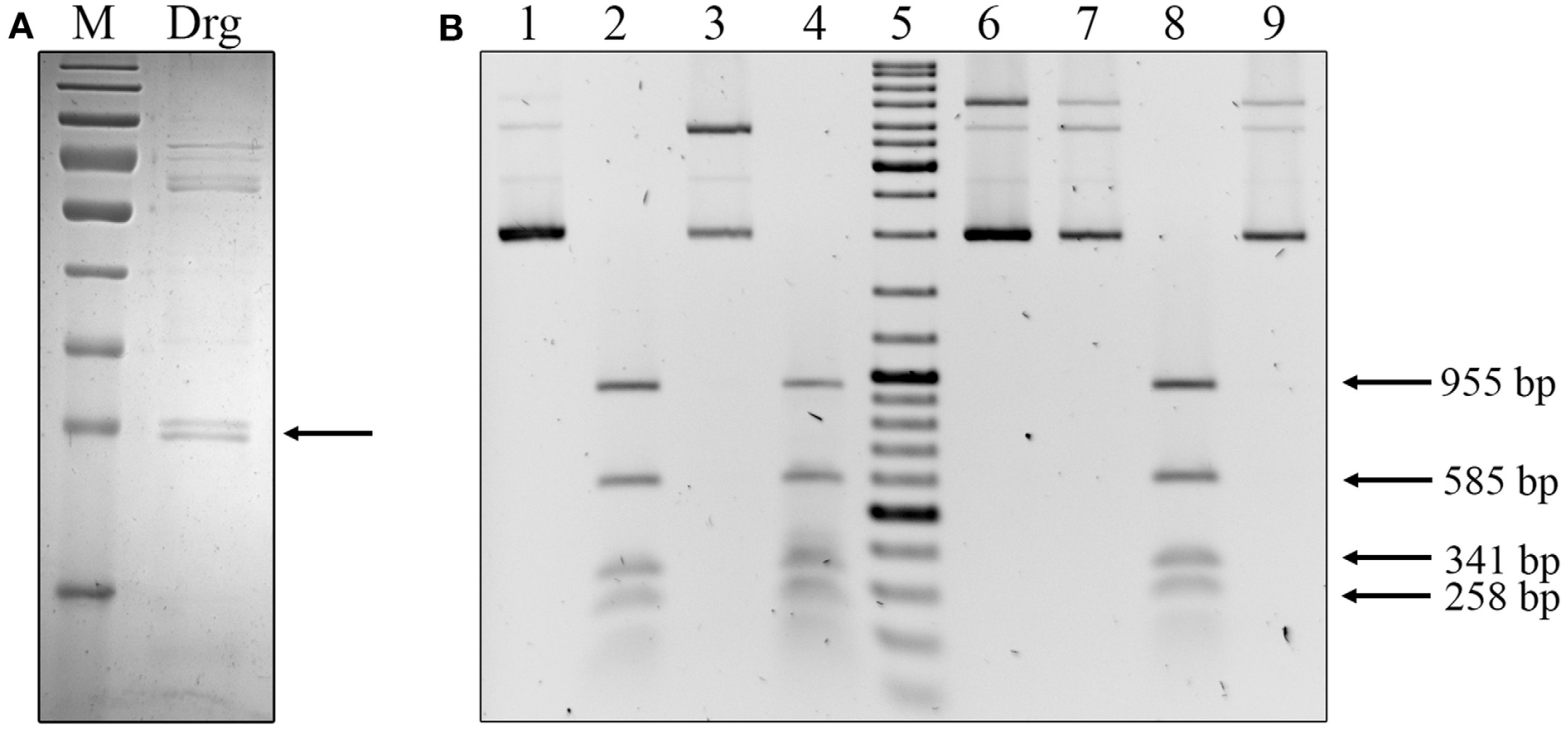
***In vitro* characterization of the *N. gonorrhoeae* FA1090 Drg endonuclease. (A)** Pre-purified Drg protein. The enzyme was separated on a 12% SDS PAGE gel and stained with Coomassie Brilliant Blue R250. M, PageRuler™ Prestained Protein Ladder (10, 15, 25, 35, 40, 55, 70, 100, 130, and 170 kDa) was used as the protein molecular mass marker. The arrow indicates the pre-purified Drg protein. **(B)** Determination of the activity and specificity of the Drg endonuclease. Lanes 1–4 methylated pUC19 DNA with GmeATC specificity. Lanes 6–9 unmethylated pUC19 DNA. 1 and 6, undigested DNA; 2 and 7, plasmids digested with DpnI (cuts only at GmeATC); 3 and 8, plasmids digested by MboI (cuts only at GATC); 4 and 9, plasmids digested by the pre-purified Drg endonuclease from *N. gonorrhoeae* FA1090; 5, GeneRuler™ DNA Ladder Mix: 10,000, 8000, 6000, 5000, 4000, 3500, 3000, 2500, 2000, 1500, 1200, 1000, 900, 800, 700, 600, 500, 400, 300, 200, and 100 bp.

To characterize the gonococcal Drg endonuclease, the *ngo0007* gene was cloned into the pET28a vector and protein encoded by the gene was pre-purified from GM2163 (T7) (pET28a::*drg*) cells using metal affinity chromatography and a Ni-NTA Agarose column. The molecular mass of Drg was *M*_(*r*)_ = 38000 ± 1000 Da as estimated from SDS-PAGE (Figure 9A) and was consistent with that predicted on the basis of the amino acid sequence.

Drg protein from *N. meningitidis* B MC58 recognizes the GATC sequence and digest only methylated DNA (Cantalupo et al., 2001). We assumed that the gonococcal Drg protein should also recognize and cleave the GmeATC sequence. Therefore, to study the activity and specificity of the gonococcal Drg endonuclease, we used pUC19 (*dam*^+^) and pUC19 (*dam*^−^) plasmids. After digestion of pUC19 (*dam*^+^) by the studied endonuclease, 15 DNA restriction fragments should be observed, four of them greater than 250 bp: 955, 585, 341, and 258. Additionally, we compared the Drg-derived DNA fragments resulting from the cleavage of pUC19 (*dam*^+^) with DpnI-digestion products, which also recognizes the GmeATC sequence. Obtained restriction patterns after digestion with Drg or DpnI were identical (Figure 9B). Moreover, as demonstrated in Figure 9B, the Drg protein cleaved only methylated DNA, while unmethylated DNA remained undigested. Based on these results, we concluded that the gonococcal Drg endonuclease is active and recognizes the GmeATC sequence.

## Discussion

In prokaryotes, DNA methylation has an important impact on the regulation of gene expression and other critical cellular processes, such as DNA repair, DNA replication and phase variation (Marinus and Casadesus, 2009). Dam methylase, which recognizes and methylates adenine in GATC sequences, plays a major role in this processes. It has been demonstrated that in several *N. meningitidis* and *N. lactamica* genomes, the *drg* gene is inserted into the *dam* locus (Cantalupo et al., 2001; Jolley et al., 2004). For the first time, we report such situation in gonococci. In the present work, we demonstrate that the *ngo0007* gene of *N. gonorrhoeae* FA1090 encodes a functional DNA endonuclease, Drg, which specifically recognizes and cuts the GmeATC sequences, similarly as DpnI (Lacks et al., 1974). The *drg* gene seems to have been acquired by *Neisseriaceae* from an exogenous source (Jolley et al., 2004), and has been inserted into the *leuS*/*dam* locus, which represents a hot spot for DNA recombination in this bacterial group. The *drg* locus in *N. gonorrhoeae* FA1090 has a lower GC-pairs content (40.5%) compared to the whole genome of *N. gonorrhoeae* FA1090 (52%) or the *leuS* locus (59.3%). The integrity of *drg*-like locus (NGFG00144) and neighboring genes (*leuS* and a methylase) was described as crucial in *N. gonorrhoeae* MS11 (Remmele et al., 2014).

DNA methylation in Proteobacteria is involved in the regulation of interactions between DNA—binding proteins and specific recognition of DNA sequences. Methylation of adenine reduces the thermodynamic stability of the DNA and alters the curvature of the DNA helix (Guo et al., 1995). These structural changes may affect the effectiveness of protein interactions with target nucleic acid sequences. Generally, the presence of methylated adenine in the DNA sequence marks the spot of potential protein-DNA interaction.

In the present work, we have demonstrated that in the *drg*-deficient strain (*N. gonorrhoeae drg::cm* mutant) certain genes are differently expressed compared to the wt *N. gonorrhoeae* strain (possessing the *drg* gene). However, the mechanism of action of the Drg protein on gene expression remains unclear. Therefore, a better understanding of the biological role of DNA endonucleases, not only in the context of DNA digestion, is necessary.

Some authors suggest that the Drg protein does not have a direct mechanistic involvement in virulence (Jolley et al., 2004). Our examination of certain features of the gonococcal *drg::cm* mutant indicates that this opinion should be revised. One of the virulence factors of *N. gonorrhoeae* is biofilm production during cervical infection (Greiner et al., 2005; Steichen et al., 2008). We have demonstrated that the absence of the *drg* gene has an impact on the first steps of gonococcal pathogenesis, such as biofilm formation. As shown by microscopy and adherence assays, the biofilm formed by the *N. gonorrhoeae drg::cm* mutant is weaker than the one formed by the parental strain. Our analysis of the *N. gonorrhoeae drg::cm* mutant transcriptome pointed out several genes encoding proteins which we grouped into COGs. Clustering of proteins permitted us to find out the function of proteins encoded by differently expressed genes. Several proteins may be engaged in biofilm formation. Furthermore, among genes with deregulated expression, some encoded uncharacterized proteins, which role in the bacterial ability to form biofilm and/or viability may be unknown.

Lipopolysaccharide (LPS) is the major surface component of Gram-negative bacteria and its polysaccharide fragment is situated at the outermost region. The important role of LPS in biofilm production has been demonstrated for various bacteria. For example, the lack of O-polysaccharide enhanced biofilm formation by *Bradyrhizobium japonicum* (Lee et al., 2010). Similar observations were made for *E. coli*, in which biofilm formation by a LPS-mutant was strongly enhanced in comparison to the parental strain. This *E. coli* mutant strain also showed an increased auto-aggregation phenotype and stronger cell surface hydrophobicity compared to the wt strain (Nakao et al., 2012). *N. gonorrhoeae* has an unusual LPS, lipooligosaccharide (LOS), consisting of a core polysaccharide and lipid A. In case of the gonococcal *drg::cm* mutant, at least two proteins, which genes were up-regulated, belong to “Cell wall/membrane/envelop biogenesis” (M category) COG and are engaged in LOS biogenesis. The first one, NGO1898 is a glucose-1-phosphate thymidylyltransferase RfbA involved in biosynthesis of the first precursor of LPS, L-rhamnose. The second protein, NGO0178, is uncharacterized, but belongs to a group of proteins involved in lipid A biosynthesis (COG3307). Other up-regulated genes in the gonococcal *drg::cm* mutant, which protein products are involved in synthesis of extracellular polysaccharides, are *ngo0207* and *ngo0220*. NGO0207 (WcaA) is homologous to a sugar transferase, required for colonic acid synthesis, involved in capsule and polysaccharide biogenesis in *Caulobacter crescentus* and *E. coli* (Patel et al., 2012). The second, NGO0220, is a potential UTP-glucose-1-phosphate uridylyltransferase GalU also involved in the colonic acid pathway.

Among proteins encoded by down-regulated genes in the *N. gonorrhoeae drg::cm* mutant only one seemed to be involved in LOS biosynthesis: NGO1765—the glycosyl transferase RfaG. *E. coli* K12 cells with mutation within the *rfaG* gene had an altered LPS biosynthesis, and their biofilm exhibited increased cell—cell interaction (Agladze et al., 2005). This is not the case for the *N. gonorrhoeae drg::cm* mutant as was shown by microscopy or adherence assays. For the second studied gonococcal mutant (*drg::dam*), both genes that encode proteins classified to COG0438 (glycosyl transferase), *ngo1765* and *ngo0418*, were down-regulated. This gonococcal *drg::dam* mutant exhibited a cemented-type of biofilm as seen on FE SEM images.

Several other genes which expression was altered in both mutants may be involved in biofilm formation. Indeed, by expression microarray analysis Falsetta et al. (2009), identified 83 differentially regulated genes by comparing biofilm-forming *N. gonorrhoeae* cells to planktonic growth. Among these genes, expression of *aniA, ccp*, and *norB* seemed to be essential for mature biofilm formation. Overall, biofilm production appeared to be an adaptation to environmental stress in the female urogenital tract. In our case, among the genes described by Falsetta, 10 were also deregulated in the *drg::cm* mutant, which had an impaired viability (planktonic growth and biofilm formation), and 14 in the *drg::dam* mutant, which ability of biofilm production and adhesion to human cells was impaired. The vast majority of these genes, deregulated in gonococcal *drg::cm* or *drg::dam* mutants, encode uncharacterized proteins. Some of deregulated genes encode proteins with known functions, e.g., expression of several genes of *nuo* operon encoding different NADH dehydrogenases, involved in respiration, was perturbed (Supplementary Material, Tables 2, 3).

Biofilm production strongly depends on the bacterial ability to form microcolonies, which results from efficient gonococcal adhesion to host cells. Microcolonies are formed when a single Opa protein binds to bacterial LOS of the neighboring bacteria (Hung and Christodoulides, 2013). These proteins also play an important role in initial colonization by mediating intimate adhesion of gonococci to epithelial cells via interactions with heparin sulfate proteoglycans and members of the carcinoembryonic antigen cell adhesion molecule (CEACAM) family (Moore et al., 2005). In a similar way, Opa proteins influence gonococcal attachment to human fallopian tube tissues (Dekker et al., 1990). The *ngo1513* gene, up-regulated in both mutants, encodes the outer membrane opacity protein D, OpaD. This protein may be responsible for altered biofilm phenotype in the studied mutants.

Several reports demonstrate that Dam activity may influence not only biofilm formation but also pathogen—target cell interactions (reviewed by Marinus and Casadesus, 2009). In our study, the gonococcal *dam*-expressing mutant had a reduced adherence to human Hec-1-B cells. Knockout of a putative *dam* gene in *Campylobacter jejuni* is accompanied, among other factors, by hyperadherence of the mutant strain to epithelial cells (Kim et al., 2008). It was demonstrated that overproduction of several bacterial Dam methylases caused an attenuated invasion in animal models (Julio et al., 2001; Chen et al., 2003). For example, Dam overproduction in *Yersinia entercolitica* strains was attenuated in mouse models (Julio et al., 2001, 2002), which was found to be associated with changes in the composition of the LPS O-antigen and transcriptional alterations of invasin genes (Fälker et al., 2007). In the studied mutants, also proteins that did not match known COGs may be involved in bacterial adhesion to target cells. In the *N. gonorrhoeae drg::cm* mutant, 3 proteins: NGO01068, NGO1393 and NGO1585, may be part of the Maf adhesion protein family. The *maf* genes encode a family of variable lipoproteins with multiple silent loci, originally identified as human glycolipid receptors-binding proteins in pathogenic *Neisseria* (Paruchuri et al., 1990; Naumann et al., 1999). All genes encoding these proteins were up-regulated in the gonococcal *drg::cm* mutant, except NGO1068. 2 genes, *ngo1068* and *ngo1585*, were also up-regulated in the *N. gonorrhoeae drg::dam* mutant. We cannot exclude that some proteins with known functions are engaged in several, totally different, cellular processes. In both mutants, expression of several genes encoding proteins that form 30S or 50S ribosomal subunits was deregulated. Such proteins are not only involved in constitution of ribosomes and the process of translation, but may also play part in recognition of endometrial cells and have a potential role in gonococcal invasion (Spence and Clark, 2000).

The *N. gonorrhoeae drg::cm* mutant is not only disabled in biofilm production, but also in its viability, as shown by cell growth assays (ATP measurement assay). The gonococcal viability may be influenced by a range of different genes. We noticed that this mutant exhibited lower expression of the *ngo1767* gene, encoding the KatE catalase. The KatE protein plays a role in peroxide stress resistance. Mutation in a similar *katA* gene in *Staphylococcus aureus* leads to a severe growth defect, attributable to the lack of catalase activity, under aerobic conditions in defined media. This causes the inability to scavenge exogenous or endogenously produced H_2_O_2_, resulting in accumulation of H_2_O_2_ in the medium. In turn, this leads to DNA damage and induces the growth defect (Cosgrove et al., 2007). Bacterial viability could also be influenced by the DNA repair systems. In bacteria, high-fidelity repair is balanced with low-fidelity repair and mutagenesis. Such balance is important for maintaining viability, while providing an opportunity for the advantageous selection of mutations when faced with a changing environment. Our transcriptome analysis showed that among the up-regulated genes in the gonococcal *drg::cm* mutant several encode proteins involved in DNA repair, such as NGO0777 (HimA), engaged in DNA site-specific recombination. This protein may be involved in DNA repair as its gene expression in *E. coli* is induced by treatment of cells with UV or mitomycin C, suggesting control by the inducible DNA repair (SOS) system (Miller et al., 1981). A second protein involved in DNA repair is NGO1930, also known as the DNA mismatch repair protein MutS (Criss et al., 2010). Another protein, which gene expression was upregulated is NGO1207–excinuclease UvrA, involved in the Nucleotide Excision Repair system (LeCuyer et al., 2010). Expression of other genes, which proteins are involved in DNA repair, was repressed in the *N. gonorrhoeae drg::cm* mutant: (i) NGO0786 is an uracil-DNA glycosylase, which prevents mutagenesis by eliminating uracil from DNA molecules initiating the Base-Excision Repair pathway (Krokan et al., 1997), (ii) NGO1173 is a DNA mismatch endonuclease Vsr (V.NgoAXIV), belongs to the Very Short Patch repair system in *N. gonorrhoeae* (Kwiatek et al., 2010). Failure to repair DNA or to the contrary—too elevated rate of DNA repair may increase the level of mutagenesis and affect the *drg::cm* mutant viability.

In conclusion, our results provide evidence that, molecular rearrangement at the *dam/drg* locus affects transcription patterns and phenotype of *N. gonorrhoeae* FA1090. These changes include the transcription of genes associated with pathogenic potential in biofilm production, adherence to human epithelial cells or bacterial cell viability.

The role of DNA methylation in control of gene expression is well documented (Marinus and Casadesus, 2009), whereas the role of endonucleases remains almost unknown. It was only demonstrated that a multifunctional eukaryotic endonuclease APE-1/Ref-1 regulates the DNA-binding activity of a number of transcription factors in a redox-dependent manner by the reduction of conserved cysteine residues in their DNA binding domains (Ando et al., 2008). This is the first report describing a potential role of an endonuclease in gene expression in bacteria. Drg may modulate transcription factors activity or even act as a transcription factor. The C-terminal domain of Drg is identical in 77% to winged helix domain of R.DpnI endonuclease from *S. pneumoniae*. This domain seems bind DNA in a sequence- and methylation-sensitive manner (Siwek et al., 2012). Via a winged helix domain, the Drg protein may sterically occupy promoter or operator sites and mediate DNA looping. DNA looping is involved in regulation of transcriptional initiation in many prokaryotic operons (Matthews, 1992). However, the exact mechanism of action of this protein remains to be determined.

### Conflict of interest statement

The authors declare that the research was conducted in the absence of any commercial or financial relationships that could be construed as a potential conflict of interest.

## Abbreviations

*drg*: *dam replacing gene*
wt: wild-type
*cm*: chloramphenicol casette
COG: clusters of orthologous groups
FE SEM: Field Emission Scanning Electron Microscopy
SCLM: Scanning Confocal Laser Microscopy
GmeATC: GATC sequence with methylated adenine.
