# Supplementary material for: The dam replacing gene product enhances Neisseria gonorrhoeae FA1090 viability and biofilm formation
Source: Front Microbiol. 2014 Dec 17;5:712. doi: 10.3389/fmicb.2014.00712 (PMC4269198; doi:10.3389/fmicb.2014.00712)
Supplement: Supplementary file 1 [file Table1.DOCX]

**Table S1. List of primers used for gene amplification or qRT-PCR.** For qRT-PCR primers, primer names are the same as gene accession number.

| **Primers for amplification of *drg* from *N. gonorrhoeae* FA1090** | | | |
| --- | --- | --- | --- |
| **Name** | **Forward primer** | **Reverse primer** | **Product size bp** |
| DrgFL | AACTGCAGAATGTTGACGAACCGTTTGAACGCCTGCTCAC | GGCAAGCTTCTCATCTACTGAACCGGGAGTTTGTTGG | 3237 |
| **Primers for amplification of *dam* from *N. meningitides* FAM18** | | | |
| **Name** | **Forward primer** | **Reverse primer** | **Product size bp** |
| Dam18 | CAACGCCTGTTGTGCCGCTGCCCATGAAAGG | CCGCAAGAACTGATTAACGCCTACGGCGCGGAC | 2465 |
| **Primers for amplification of *drg* (cloning into pET28)** | | | |
| **Name** | **Forward primer** | **Reverse primer** | **Product size bp** |
| DrgpET | CGGCGCTAGCAATTTATTTTTCGATACCCAATTGG | CGGAAGCTTTCAATCATAAATTGCCAATAATTCTC | 946 |
| **Primers for qRT-PCR** | | | |
| **Primers for internal standard** | | | |
| **Name** | **Forward primer** | **Reverse primer** | **Product size bp** |
| 16SRNA | gcgtgggtagcaaacaggat | CGCGTTAGCTACGCTACCAAG | 81 |
| **Primers for the study *drg::cm* mutant gene expression** | | | |
| **Name** | **Forward primer** | **Reverse primer** | **Product size bp** |
| Ngo0007 | AATGGCAATCCAACCTGTTT | CGACAATGTGAATTCTTCGG | 107 |
| Ngo1068 | GGAATAAACCGCTGGAATCA | GCATCTACTGATTGCGGAAA | 130 |
| Ngo1586 | CTGATGATTTGCGCCCTAT | AAACCAACTCATTGAACAAATCA | 124 |
| Ngo1589 | ATGCAGCAGCAAAGAAATTG | TGAAGATGCTTCTTTCCAAGC | 140 |
| Ngo1592 | GCCGTTATTGATTTGAATAGGA | TTTGGCTGGAATTGTTTCAA | 81 |
| Ngo1648 | CAAAGACACCGCATCAGTTC | CGCTTGTCCCTGATTGTATG | 116 |
| Ngo1767 | TTCGGTTCGCATACCTACAG | AGCTTCTTCGTTGGTCAGGT | 108 |
| Ngo2093 | ACCAACATCGTTACGCTTCA | GTCGACAGAGTTCTGACCCA | 150 |
| Ngo2094 | TGACCATCCGTCCTTTACAC | CGATGACTTCGCCCATATC | 123 |
| **Primers for the study *drg::dam* mutant gene expression** | | | |
| **Name** | **Forward primer** | **Reverse primer** | **Product size bp** |
| Ngo0095 | TGCCGACATAACGCATATTT | CAGCTTACAGGGCCGAAC | 133 |
| Ngo0206 | ATCAACACCTTGGCAATCAA | ACACCAAATCCCACTCGTTT | 85 |
| Ngo0207 | TGAAAGGATTGTGCCGATT | TGCCGCATTAAATAACCTCA | 129 |
| Ngo0277 | AATCTGGGCATCTTTATCGG | ACGGTTTCATTAGGTTTGGC | 69 |
| Ngo0365 | GCAATCGGGAATAGTTGGAT | TGGTTGTGAAACTGTTTGGG | 133 |
| Ngo0406 | GATATGCAAAGCAAAGCGAA | TGCATTTCTTTCATTTGTCCA | 131 |
| Ngo0574 | gtcttccatctggaacgtca | CGGCAAAGCGGTAGTATTTC | 71 |
| Ngo0640 | TGCTCATCGTGCGTGTAATA | TGCGTTTGAAGGTAAACAGC | 102 |
| Ngo0675 | AGATTAATTGCCCACCCATC | TTCGGTTACGATATGGACGA | 104 |
| Ngo0676 | AAAGGGCTGCTTTCAGTAGG | CAGGAAGCGCATTTATCTGA | 123 |
| Ngo0853 | TCACTGGAAACCGTAACCCT | ATTTGACTGCCGTCCCTATC | 127 |
| Ngo0869 | ATCCTGCATATCGACCAACA | AAGGGCGTAACAATCAGACC | 113 |
| Ngo0874 | CCGCACATTGTTGTCGTAA | CCCTGTACCCAAGGCAATAG | 68 |
| Ngo0906 | TGACCGACGAAGAAGAGTTG | TTCACGCCCAACTTAGTCAG | 124 |
| Ngo1068 | GGAATAAACCGCTGGAATCA | GCATCTACTGATTGCGGAAA | 130 |
| Ngo1368 | TTATGGCGACGGTAATCAAA | ACCCAAATGCAGAATAAGGC | 118 |
| Ngo1368 | TTATTGCTGATTGCCTCTGC | AGGCTGACAACGTGAATCAA | 113 |
| Ngo1435 | TGGGTGTTGGGTTTATTAGGTT | ACCAAAGCAACAATACCGACT | 143 |
| Ngo1484 | TCGATGCCGTACTCAAAGAC | TTCAATGACTTCAGCTTGGC | 137 |
| Ngo1506 | ATAACGTGCAACCGTCTGTC | TGATGTTCAGGCACAAGAGG | 94 |
| Ngo1513 | CGGCAACCAGCTTAACATAA | GGGTTTGAATTTGTCGTTGA | 124 |
| Ngo1559 | ACAGACGCTGGTGCAGTATC | GCGTCTGCAGATAGTAGGCA | 131 |
| Ngo1585 | AATGATTGTCCGCCTAAACC | GGTTTCCTGTGTCGGAATCT | 144 |
| Ngo1589 | ATGCAGCAGCAAAGAAATTG | TGAAGATGCTTCTTTCCAAGC | 140 |
| Ngo1775 | GATATGAAATACGGCAGCGA | GCTTCCTGTTTGGCTTTGTA | 77 |
| Ngo1991 | AATATTGGCGGGAATGTTTC | CATTTCCCTTCCTGTTAGCC | 111 |
| Ngo2093 | ACCAACATCGTTACGCTTCA | GTCGACAGAGTTCTGACCCA | 150 |
| Ngo2127 | TGTTAGGAGATTCCGATGGAG | TATTGTCAGCGCCACAACTT | 114 |
