# Supplementary material for: The dam replacing gene product enhances Neisseria gonorrhoeae FA1090 viability and biofilm formation
Source: Front Microbiol. 2014 Dec 17;5:712. doi: 10.3389/fmicb.2014.00712 (PMC4269198; doi:10.3389/fmicb.2014.00712)
Supplement: Supplementary file 2 [file Table2.DOCX]

**Table S2. Differentially expressed genes in the *drg-*deficient *N. gonorrhoeae* mutant versus wild-type *N. gonorrhoeae* FA1090.** Identity of the genes is indicated by gene and protein accession numbers, according to NCBI in the annotation of the *N. gonorrhoeae* FA1090 genome. For microarray and qRT-PCR values, the presented average ratio is the mean of *N. gonorrhoeae drg::cm* mutant: wild type *N. gonorrhoeae* FA1090. Only those genes with an expression ratio above 1.5-fold and *P* < 0.05 were included in this study. Protein functions were assigned according to BLAST and Uniprot databases. COGs were assigned with the NCBI Conserved domains server (http://www.ncbi.nlm.nih.gov/Structure/cdd/wrpsb.cgi). NF – known conserved domain not found.

| Gene  Protein | Function | Microarray analysis (change fold) | qRT-PCR (change fold) | COG (category) |
| --- | --- | --- | --- | --- |
| **up** |  |  |  |  |
| NGO0007  YP_207191.1 | Putative type II restriction endonuclease | 2.58^[[1]](#footnote-1)^ | 0.83^[[2]](#footnote-2)^ | COG0338 (L) |
| NGO0009  YP_207193.1 | Hypothetical protein | 1.77 |  | NF |
| NGO0024  YP_207205.1 | Hypothetical protein | 2.16 |  | NF |
| NGO0045  YP_207224.1 | Acetyl-CoA carboxylase biotin carboxyl carrier protein subunit AccB | 1.67 |  | COG0511 (I) |
| NGO0115  YP_207287.1 | Hypothetical protein | 2.05 |  | NF |
| NGO0116  YP_207288.1 | Preprotein translocase subunit SecB | 1.88 |  | COG1952 (U) |
| NGO0122  YP_207294.1 | Hypothetical protein | 1.91 |  | NF |
| NGO0137  YP_207307.1 | Hypothetical protein | 1.63 |  | NF |
| NGO0160  YP_207328.1 | Hypothetical protein | 1.77 |  | NF |
| NGO0161  YP_207329.1 | Hypothetical protein | 1.79 |  | NF |
| NGO0162  YP_207330.1 | Hypothetical protein | 1.91 |  | NF |
| NGO0163  YP_207331.1 | Hypothetical protein | 1.50 |  | NF |
| NGO0178  YP_207345.1 | Hypothetical protein RfaL | 1.62 |  | COG3307 (M) |
| NGO0191  YP_207358.1 | 30S ribosomal protein S15 RpsO | 2.07 |  | COG0184 (J) |
| NGO0202  YP_207367.1 | Putative phosphoenolpyruvate synthase regulatory protein | 1.56 |  | COG1806 (S) |
| NGO0207  YP_207372.1 | Sugar transferase WcaA | 1.54 |  | COG0463 (M) |
| NGO0220  YP_207385.1 | UTP-glucose-1-phosphate uridylyltransferase GalU | 1.63 |  | COG1210 (M) |
| NGO0230  YP_207395.1 | Trk system potassium uptake protein TrkG | 1.74 |  | COG0168 (P) |
| NGO0319  YP_207478.1 | Hypothetical protein DadA/ Glycine/D-amino acid oxidase | 1.54 |  | COG0665 (E) |
| NGO0351  YP_207507.1 | Glutaredoxin-like protein GrlA | 1.82 |  | COG0278 (O) |
| NGO0364  YP_207520.1 | Restriction endonuclease R.NgoAVII | 3.19 |  | NF |
| NGO0369  YP_207525.1 | Hypothetical protein | 1.89 |  | COG0607 (P) |
| NGO0376  YP_207532.1 | Peptidyl-prolyl cis-trans isomerase B PpiB | 1.89 |  | COG0652 (O) |
| NGO0406 | HsdS subunit, type I restriction-modification system NgoAV | 1.57 |  | COG0732 (V) |
| NGO0442  YP_207595.1 | 50S ribosomal protein L25 RplY | 1.55 |  | COG1825 (J) |
| NGO0464  YP_207614.1 | Hypothetical protein | 2.11 |  | NF |
| NGO0466  YP_207616.1 | Phage associated protein | 1.78 |  | NF |
| NGO0469  YP_207619.1 | Phage associated protein | 1.64 |  | NF |
| NGO0474  YP_207624.1 | Phage associated protein | 2.10 |  | NF |
| NGO0482  YP_207632.1 | Phage associated protein | 1.84 |  | NF |
| NGO0490  YP_207640.1 | Phage associated protein | 1.78 |  | NF |
| NGO0491  YP_207641.1 | Phage associated protein | 1.73 |  | NF |
| NGO0510  YP_207660.1 | Phage associated protein | 2.14 |  | COG3210 (U) |
| NGO0563  YP_207708.1 | Hypothetical protein | 1.87 |  | NF |
| NGO0574  YP_207719.1 | Carbonic anhydrase Cah | 2.20 |  | COG3338 (P) |
| NGO0615  YP_207754.2 | Ribonucleotide-diphosphate reductase subunit beta nrdB | 1.51 |  | COG0208 (F) |
| NGO0621  YP_207761.1 | Hypothetical protein | 1.56 |  | COG4867 (R) |
| NGO0622  YP_207762.1 | Hypothetical protein | 1.85 |  | NF |
| NGO0638  YP_207777.1 | Hypothetical protein | 2.39 |  | NF |
| NGO0676  YP_207815.1 | Cytosine-specific Type II DNA methyltransferase NgoAXV | 1.53 |  | COG0270 (L) |
| NGO0698  YP_207834.1 | Virulence_RhuM super family | 1.68 |  | COG3943 (R) |
| NGO0699  YP_207835.1 | Hypothetical protein | 1.89 |  | COG0732 (V) |
| NGO0700  YP_207836.1 | Hypothetical protein | 1.96 |  | NF |
| NGO0701  YP_207837.1 | Hypothetical protein | 1.76 |  | NF |
| NGO0712  YP_207844.1 | Hypothetical protein | 1.86 |  | NF |
| NGO0777  YP_207903.1 | DNA-binding protein Hu HimA | 1.89 |  | COG0776 (L) |
| NGO0806  YP_207932.1 | Hypothetical protein | 1.68 |  | NF |
| NGO0816  YP_207940.1 | Hypothetical protein | 1.61 |  | NF |
| NGO0869  YP_207982.1 | Hypothetical protein/DedA protein ortholog/uncharacterized membrane-associated protein | 1.84 |  | COG0586 (S) |
| NGO0874  YP_207987.1 | Type II DNA restriction endonuclease R.NgoAIV | 1.62 |  | NF |
| NGO0880  YP_207992.1 | Hypothetical protein | 1.52 |  | COG2840 (S) |
| NGO0894  YP_208005.1 | tRNA-dihydrouridine synthase | 1.57 |  | COG0042 (J) |
| NGO0907  YP_208017.1 | Trafficking protein B (FitB) | 1.54 |  | COG1487 (R) |
| NGO0912  YP_208020.1 | Succinyl-CoA ligase [ADP-forming] subunit SucD | 1.68 |  | COG0074 (C) |
| NGO0916  YP_208024.1 | Dihydrolipoyllysine-residue succinyltransferase component of 2-oxoglutarate dehydrogenase complex AceF | 1.79 |  | COG0508 (C) |
| NGO0983  YP_208081.1 | Outer membrane protein H.8 Lip | 1.72 |  | NF |
| NGO0998  YP_208093.1 | DNA primase DnaG | 1.83 |  | COG0358 (L) |
| NGO1046  YP_208130.1 | Putative ClpB protein (response to heat) | 2.23 | 1.44 | COG0542 (O) |
| NGO1054  YP_208137.1 | Hypothetical protein | 1.54 |  | NF |
| NGO1073  YP_208154.1 | Hypothetical protein | 1.53 |  | NF |
| NGO1093  YP_208172.1 | Phage associated protein | 1.73 |  | NF |
| NGO1095  YP_208174.1 | Phage associated protein | 1.96 |  | NF |
| NGO1101  YP_208180.1 | Hypothetical protein | 1.54 |  | NF |
| NGO1102  YP_208181.1 | Hypothetical protein | 2.86 |  | NF |
| NGO1103  YP_208182.1 | Hypothetical protein | 2.42 |  | NF |
| NGO1111  YP_208190.1 | Hypothetical protein | 1.63 |  | NF |
| NGO1115  YP_208194.1 | Phage associated protein | 1.98 |  | NF |
| NGO1141  YP_208220.1 | Phage associated protein | 1.88 |  | NF |
| NGO1143  YP_208222.1 | Phage associated protein | 1.59 |  | NF |
| NGO1144  YP_208223.1 | Phage associated protein | 2.13 |  | NF |
| NGO1145  YP_208224.1 | Phage associated protein | 2.16 |  | NF |
| NGO1149  YP_208228.1 | O-succinyl homoserine sulfhydrolase MetC | 1.54 |  | COG0626 (E) |
| NGO1177  YP_208251.1 | Hypothetical protein PilE | 1.51 |  | COG4968 (NU) |
| NGO1184  YP_208256.1 | Hypothetical protein NemA | 0.63 |  | COG1902 (C) |
| NGO1207  YP_208278.1 | Excinuclease ABC subunit A uvrA | 1.76 |  | COG0178 (L) |
| NGO1208  YP_208279.1 | Restriction endonuclease R.NgoAIII | 1.68 |  | NF |
| NGO1209  YP_208280.1 | Cytosine-specific methyltransferase Dcm | 1.73 |  | COG0270 (L) |
| NGO1226  YP_208297.1 | Putative DNA polymerase III chi subunit, HolC | 1.52 |  | COG2927 (L) |
| NGO1234  YP_208305.1 | Hypothetical protein | 1.66 |  | COG1359 (S) |
| NGO1241  YP_208312.1 | Histidinol-phosphate aminotransferase HisC | 2.02 |  | COG0079 (E) |
| NGO1242  YP_208313.1 | Imidazoleglycerol-phosphate dehydratase HisB | 1.69 |  | COG0131 (E) |
| NGO1248  YP_208319.1 | Hypothetical protein | 2.45 |  | NF |
| NGO1249  YP_208320.1 | Hypothetical protein | 1.56 |  | COG0599 (S) |
| NGO1304  YP_208369.1 | DNA-binding competence protein 2 ComE2 | 1.56 |  | COG1555 (L) |
| NGO1367  YP_208427.1 | Hypothetical protein | 1.66 |  | NF |
| NGO1393  YP_208453.1 | Adhesin. MafA-like protein | 1.62 |  | NF |
| NGO1404  YP_208461.1 | Glycine cleavage system protein H GcvH | 1.74 |  | COG0509 (E) |
| NGO1432  YP_208487.1 | Hypothetical protein TauA | 1.64 |  | COG0715 (P) |
| NGO1448  YP_208502.1 | UDP-2.3-diacylglucosamine hydrolase | 1.54 |  | COG2908 (S) |
| NGO1472  YP_208524.1 | Pyridine nucleotide transhydrogenase pntB | 1.68 |  | COG1282 (C) |
| NGO1482  YP_208532.1 | Hypothetical protein | 1.70 |  | COG2830 (S) |
| NGO1484  YP_208534.1 | Hypothetical protein | 1.61 |  | NF |
| NGO1497  YP_208547.1 | Hypothetical protein | 1.77 |  | NF |
| NGO1513  YP_208563.1 | Outer membrane opacity protein D OpaD protein | 1.52 |  | COG3637 (M) |
| NGO1585  YP_208626.1 | MafB-like adhesin | 1.65 |  | NF |
| NGO1586  YP_208627.1 | Hypothetical protein | 1.73 | 1.2 | NF |
| NGO1588  YP_208628.1 | Hypothetical protein | 2.13 |  | NF |
| NGO1589  YP_208629.1 | Hypothetical protein/ possibly an alternative C terminus for MafB | 1.69 | 1.2 | NF |
| NGO1590  YP_208630.1 | Hypothetical protein | 2.47 |  | NF |
| NGO1591  YP_208631.1 | Hypothetical protein | 1.69 |  | NF |
| NGO1592  YP_208632.1 | Hypothetical protein McrA | 2.11 | 1.4 | COG1403 (V) |
| NGO1593  YP_208633.1 | Hypothetical protein | 2.55 |  | NF |
| NGO1594  YP_208634.1 | Hypothetical protein | 1.55 |  | NF |
| NGO1595  YP_208635.1 | Hypothetical protein | 1.73 |  | NF |
| NGO1596  YP_208636.1 | Hypothetical protein | 1.87 |  | NF |
| NGO1601  YP_208641.1 | Hypothetical protein | 1.51 |  | NF |
| NGO1610  YP_208650.1 | Transaldolase MipB | 1.52 |  | COG0176 (G) |
| NGO1639  YP_208679.1 | Phage associated protein | 1.85 |  | NF |
| NGO1676  YP_208713.1 | 50S ribosomal protein L21 RplU | 1.62 |  | COG0261 (J) |
| NGO1684  YP_208721.1 | NADPH-dependent 7-cyano-7-deazaguanine reductase | 1.54 |  | COG0780 (R) |
| NGO1695  YP_208731.1 | Phospho-2-dehydro-3-deoxyheptonate aldolase AroG | 1.51 |  | COG0722 (E) |
| NGO1709  YP_208742.1 | Hypothetical protein | 1.71 |  | COG2979 (S) |
| NGO1739  YP_208772.1 | Catalytic domain of type II restriction endonucleases | 1.73 |  | COG3886 (L) |
| NGO1740  YP_208773.1 | NADH dehydrogenase subunit L, NuoL | 1.54 |  | COG1009 (CP) |
| NGO1742  YP_208775.1 | Putative NADH dehydrogenase I chain J NuoJ | 1.60 |  | COG0839 (C) |
| NGO1759  YP_208792.1 | Hypothetical protein | 3.14 |  | NF |
| NGO1805  YP_208835.1 | Hypothetical protein | 1.54 |  | NF |
| NGO1812  YP_208842.1 | Major outer membrane protein porin P.IB OmpC | 1.54 |  | COG3203 (M) |
| NGO1817  YP_208846.1 | 50S ribosomal protein L17 rplQ | 1.59 |  | COG0203 (J) |
| NGO1820  YP_208849.1 | 30S ribosomal protein S11 RpsK | 1.70 |  | COG0100 (J) |
| NGO1821  YP_208850.1 | 30S ribosomal protein S13 rpsM | 1.71 |  | COG0099 (J) |
| NGO1839  YP_208873.1 | Hypothetical protein | 2.14 |  | NF |
| NGO18781  YP_208911.1 | Hypothetical protein | 2.29 |  | NF |
| NGO1898  YP_208926.1 | Glucose-1-phosphate thymidylyltransferase RfbA | 1.61 |  | COG1209 (M) |
| NGO1929  YP_208954.1 | Hypothetical protein | 1.80 |  | NF |
| NGO1930  YP_208955.1 | DNA mismatch repair protein MutS MutS | 1.81 |  | COG0249 (L) |
| NGO1969  YP_208990.1 | Hypothetical protein | 1.73 |  | NF |
| NGO1970  YP_208991.1 | Hypothetical protein | 3.42 |  | NF |
| NGO1976  YP_208997.1 | Hypothetical protein | 2.34 |  | NF |
| NGO1981  YP_209001.1 | Hypothetical protein | 1.53 |  | NF |
| NGO2026  YP_209046.1 | Hypothetical protein | 1.55 |  | NF |
| NGO2055  YP_209074.1 | Hydrolase | 1.50 |  | COG1011 (R) |
| NGO2090  YP_209103.1 | ABC transporter permease. enterobactin CeuC | 1.80 |  | COG4605 (P) |
| NGO2094  YP_209107.1 | Co-chaperonin GroES | 2.47 | 2.03 | COG0234 (O) |
| NGO2134  YP_209143.1 | 30S ribosomal protein S21 RpsU | 1.89 |  | COG0828 (J) |
| NGO2173  YP_209178.1 | 50S ribosomal protein L32 RpmF | 1.94 |  | COG0333 (J) |

*not statistically significant

| Gene  Protein | Function | Microarray analysis (change fold) | qRT-PCR (change fold) | COG (category) |
| --- | --- | --- | --- | --- |
| **down** |  |  |  |  |
| NGO0023  YP_207204.1 | ABC transporter substrate-binding protein CeuA. iron related | 0.63 |  | COG4607 (P) |
| NGO0025  YP_207206.1 | AraC family transcriptional regulator | 0.44 |  | COG2207 (K) |
| NGO0076  YP_207249.1 | Hypothetical protein | 0.60 |  | COG2819 (R) |
| NGO0102  YP_207275.1 | Putative cytochrome biogenesis protein ResB | 0.66 |  | COG1333 (O) |
| NGO0109  YP_207282.1 | Hypothetical protein | 0.56 |  | NF |
| NGO0167  YP_207335.1 | Hypothetical protein | 0.36 |  | NF |
| NGO0223  YP_207388.1 | Inorganic pyrophosphatase Ppa | 0.66 |  | COG0221 (C) |
| NGO0387  YP_207543.1 | GTP cyclohydrolase FolE2 | 0.63 |  | COG1469 (S) |
| NGO0486  YP_207636.1 | Phage associated protein | 0.58 |  | NF |
| NGO0494  YP_207644.1 | Hypothetical protein | 0.55 |  | NF |
| NGO0498  YP_207648.1 | Hypothetical protein | 0.54 |  | NF |
| NGO0503  YP_207653.1 | Phage associated protein | 0.42 |  | NF |
| NGO0531  YP_207681.1 | Hypothetical protein | 0.50 |  | NF |
| NGO0552  YP_207698.1 | Permeases of the drug/metabolite transporter RhaT | 0.63 |  | COG0697 (GER) |
| NGO0555  YP_207701.1 | Hypothetical protein | 0.36 |  | COG4783 (R) |
| NGO0725  YP_207857.1 | Phage associated protein | 0.44 |  | NF |
| NGO0727  YP_207858.1 | Baseplate protein. phage associated protein | 0.59 |  | COG3948 (R) |
| NGO0731  YP_207862.1 | Phage associated protein | 0.63 |  | NF |
| NGO0786  YP_207912.1 | Uracil-DNA glycosylase Ung | 0.65 |  | COG0692 (L) |
| NGO0796  YP_207922.1 | Hypothetical protein | 0.57 |  | NF |
| NGO0836  YP_207957.1 | Hypothetical protein | 0.47 |  | NF |
| NGO0895  YP_208006.1 | Hypothetical protein | 0.66 |  | NF |
| NGO0955  YP_208056.1 | Hypothetical protein | 0.42 |  | NF |
| NGO0967  YP_208065.1 | Hypothetical protein | 0.60 |  | NF |
| NGO0982  YP_208080.1 | Hypothetical protein | 0.61 |  | NF |
| NGO1000  YP_208095.1 | Phage associated protein/ CRISPR/Cas system-associated protein Cas4 | 0.65 |  | COG1468 (L) |
| NGO1002  YP_208097.1 | Hypothetical protein | 0.65 |  | NF |
| NGO1013  YP_208108.1 | Phage repressor. phage associated protein HipB | 0.63 |  | COG1396 (K) |
| NGO1015  YP_208110.1 | Phage associated protein | 0.51 |  | COG2378 (K) |
| NGO1040  YP_208124.1 | Hypothetical protein | 0.67 |  | NF |
| NGO1068  YP_208150.1 | MafB-like protein | 0.65 | 0.65 | NF |
| NGO1104  YP_208183.1 | Phage associated protein | 0.65 |  | NF |
| NGO1147  YP_208226.1 | Hypothetical protein | 0.58 |  | NF |
| NGO1169  YP_208247.1 | Phage associated protein | 0.67 |  | NF |
| NGO1173  YP_208249.1 | DNA mismatch endonuclease. patch repair protein Vsr | 0.61 |  | COG3727 (L) |
| NGO1257  YP_208328.1 | Hypothetical protein | 0.61 |  | NF |
| NGO1293  YP_208359.1 | Hypothetical protein | 0.60 |  | NF |
| NGO1327  YP_208389.1 | Hypothetical protein | 0.63 |  | NF |
| NGO1333  YP_208395.1 | DNA topoisomerase IV subunit B GyrB | 0.66 |  | COG0187 (L) |
| NGO1427  YP_208482.1 | Transcriptional regulator repressor | 0.58 |  | COG2932 (K) |
| NGO1431  YP_208486.1 | Hypothetical protein CirA | 0.66 |  | COG1629 (P) |
| NGO1444  YP_208498.1 | Hypothetical protein | 0.65 |  | NF |
| NGO1455  YP_208508.1 | Hypothetical protein MntH | 0.66 |  | COG1914 (P) |
| NGO1576  YP_208617.1 | Hypothetical protein | 0.64 |  | NF |
| NGO1637  YP_208677.1 | Phage associated protein | 0.41 |  | NF |
| NGO1648  YP_208687.1 | Invertase related gene 7/ phage associated protein | 0.62 | 0.55 | COG3547 (L) |
| NGO1653  YP_208692.1 | Hypothetical protein | 0.65 |  | NF |
| NGO1751  YP_208784.1 | NADH-quinone oxidoreductase subunit A NuoA | 0.61 |  | COG0838 (C) |
| NGO1765  YP_208797.1 | Glycosyltransferase RfaG | 0.63 |  | COG0438 (M) |
| NGO1767  YP_208798.1 | Catalase KatE | 0.53 | 0.26 | COG0753 (P) |
| NGO1768  YP_208799.1 | Putative manganese efflux pump MntP | 0.62 |  | COG1971 (S) |
| NGO1771  YP_208802.1 | Hypothetical protein MscS | 0.30 |  | COG0668 (M) |
| NGO1778  YP_208809.1 | Leucyl/phenylalanyl-tRNA--protein transferase Aat | 0.65 |  | COG2360 (O) |
| NGO1783  YP_208814.1 | Pseudouridine synthase protein RluA | 0.64 |  | COG0564 (J) |
| NGO1847  YP_208880.1 | Hypothetical protein | 0.48 |  | NF |
| NGO1957  YP_208980.1 | Serine/threonine transporter SstT | 0.67 |  | COG3633 (E) |
| NGO2059  YP_209078.1 | Putative peptide methionine sulfoxide reductase MsrA | 0.62 |  | COG0225 (O) |
| NGO2093  YP_209106.1 | Ferric enterobactin receptor CirA, FetA | 0.56 | 0.45 | COG1629 (P) |
| NGO2118  YP_209128.1 | Hypothetical protein Ttg2C | 0.66 |  | COG1463 (Q) |

1. Overexpression corresponds only to 3`remains of *drg* gene, which probably results from run-off transcription of the chloramphenicol cassette. [↑](#footnote-ref-1)
2. Not statistically significant *P* > 0.05 [↑](#footnote-ref-2)
