# Supplementary material for: The dam replacing gene product enhances Neisseria gonorrhoeae FA1090 viability and biofilm formation
Source: Front Microbiol. 2014 Dec 17;5:712. doi: 10.3389/fmicb.2014.00712 (PMC4269198; doi:10.3389/fmicb.2014.00712)
Supplement: Supplementary file 3 [file Table3.DOCX]

**Table S3. Differentially expressed genes in *N. gonorrhoeae* with inserted *dam* gene versus wild-type *N. gonorrhoeae* FA1090.** Identity of the genes is indicated by gene and protein accession numbers according to NCBI in the annotation of the *N. gonorrhoeae* FA1090 genome. For microarray and qRT-PCR values, the presented average ratio is the mean of *N. gonorrhoeae drg::dam* mutant: wild type *N. gonorrhoeae* FA1090. Only those genes with an expression ratio above 1.5-fold and *P* < 0.05 were included in this study. Protein functions were assigned according to BLAST and Uniprot databases. COGs were assigned with the NCBI Conserved domains server (http://www.ncbi.nlm.nih.gov/Structure/cdd/wrpsb.cgi). NF – known conserved domain not found.

| Gene Protein | Function | Microarray analysis (change fold) | qRT-PCR (change fold) | COG number (COG category) |
| --- | --- | --- | --- | --- |
| **up** |  |  |  |  |
| NGO0015 YP_207196.1 | Hypothetical protein | 2.53 |  | NF |
| NGO0044 YP_207223.1 | Acetyl-CoA carboxylase, biotin carboxylase | 1.63 |  | COG0439 (I) |
| NGO0095 YP_207268.1 | PilP protein | 1.9 | 4.70 | COG3168 (NU) |
| NGO0141 YP_207311.1 | Hypothetical protein | 1.72 |  | NF |
| NGO0142 YP_207312.1 | Putative sugar transporter | 1.82 |  | COG0738 (G) |
| NGO0156 YP_207326.1 | Hypothetical protein | 1.53 |  | COG3298 (L) |
| NGO0191 YP_207358.1 | 30S ribosomal protein S15, RpsO | 1.67 |  | COG0184 (J) |
| NGO0206 YP_207371.1 | ABC transporter/Putrescine-binding periplasmic protein | 1.63 | 9.00 | COG0687 (E) |
| NGO0207 YP_207372.1 | Sugar transferase WcaA | 2.14 | 7.38 | COG0463 (M) |
| NGO0220 YP_207385.1 | UTP-glucose-1-phosphate uridylyltransferase GalU | 1.63 |  | COG1210 (M) |
| NGO0229 YP_207394.1 | Hypothetical protein | 2.16 |  | NF |
| NGO0271 YP_207434.1 | Hypothetical protein | 1.58 |  | NF |
| NGO0277 YP_207439.1 | Competence lipoprotein ComL precursor |  | 2.96 | COG4105 (R) |
| NGO0307 YP_207466.1 | Uncharacterized protein FxsA (Protein affecting phage T7 exclusion by the F plasmid) | 2.64 |  | COG3030 (R) |
| NGO0330 YP_207488.1 | Hypothetical protein | 1.67 |  | NF |
| NGO0365 YP_207521.1 | Cytosine-specific DNA methyltransferase M.NgoAVII | 2.44 | 3.64 | COG0270 (L) |
| NGO0406 | HsdS subunit, type I restriction-modification system NgoAV | 2.06 | 4.25 | COG0732 (V) |
| NGO0524 YP_207674.1 | Putative integrase | 1.54 |  | COG0582 (L) |
| NGO0567 YP_207712.1 | Hydrolase MhpD | 1.61 |  | COG0179 (Q) |
| NGO0574 YP_207719.1 | Carbonic anhydrase Cah | 1.83 | 5.68 | COG3338 (P) |
| NGO0620 YP_207760.1 | Aspartate 1-decarboxylase | 1.54 |  | COG0853 (H) |
| NGO0635 YP_207774.1 | Hypothetical protein | 1.55 |  | NF |
| NGO0640 YP_207779.1 | Type III restriction modification system-Res protein, NgoAVII | 2.14 | 3.97 | COG3421 (S) |
| NGO0641 YP_207780.1 | Type III restriction/modification system modification methylase NgoAVII | 1.57 |  | COG0863 (L) |
| NGO0667 YP_207806.1 | Hypothetical protein | 2.10 |  | NF |
| NGO0675 YP_207814.1 | Type II restriction endonuclease NgoAXV | 1.63 | 5.27 | NF |
| NGO0676 YP_207815.1 | Cytosine-specific Type II DNA methyltransferase NgoAXV | 1.69 | 4.71 | COG0270 (L) |
| NGO0736 YP_207866.1 | Hypothetical protein | 1.74 |  | NF |
| NGO0757 YP_207884.1 | Hypothetical protein | 1.51 |  | COG3678 (UNTP) |
| NGO0787 YP_207913.1 | Hypothetical protein | 1.52 |  | COG1585 (OU) |
| NGO0816 YP_207940.1 | Hypothetical protein | 1.96 |  | NF |
| NGO0818 YP_207941.1 | Hypothetical protein | 1.74 |  | NF |
| NGO0865 YP_207978.1 | Hypothetical protein | 1.58 |  | NF |
| NGO0869 YP_207982.1 | Hypothetical protein/DedA protein ortholog/uncharacterized membrane-associated protein | 3.66 | 3.46 | COG0586 (S) |
| NGO0874 YP_207987.1 | Type II DNA restriction endonuclease, NgoAIV | 1.74 | 3.5 | NF |
| NGO0881 YP_207993.1 | Putative ORF B of IS150/ transposase | 1.62 |  | COG2801(L) |
| NGO0892 YP_208003.1 | Hypothetical protein | 1.55 |  | NF |
| NGO0897 YP_208008.1 | Hypothetical protein | 2.14 |  | NF |
| NGO0904 YP_208014.1 | Hypothetical protein/ Fe-S oxidoreductase | 1.98 |  | COG0247(C) |
| NGO0906 YP_208016.1 | Conserved hypothetical iron-sulfur protein/ containing a ferredoxin-like domain | 1.79 | 4.89 | COG1139 (C) |
| NGO0907 YP_208017.1 | Trafficking protein B (FitB) |  |  | COG1487 (R) |
| NGO0914 YP_208022.1 | Hypothetical protein | 1.66 |  | NF |
| NGO0917 YP_208025.1 | Putative 2-oxoglutarate dehydrogenase, E1 component SucA | 1.50 |  | COG0567 (C) |
| NGO0918 YP_208026.1 | Type II citrate synthase GltA | 1.63 |  | COG0372 (C) |
| NGO0937 YP_208044.1 | Hypothetical protein | 1.87 |  | NF |
| NGO0938 YP_208045.1 | Hypothetical protein | 2.40 |  | NF |
| NGO0948 YP_208051.1 | Hypothetical protein/lipoprotein | 1.74 |  | COG3317(M) |
| NGO0983 YP_208081.1 | Outer membrane protein H.8 (Lip) | 1.62 |  | NF |
| NGO1010 YP_208105.1 | Phage asociated protein | 1.75 |  | NF |
| NGO1011 YP_208106.1 | Phage asociated protein | 2.02 |  | NF |
| NGO1012 YP_208107.1 | Phage asociated protein | 1.64 |  | NF |
| NGO1054 YP_208137.1 | Hypothetical protein | 2.00 |  | NF |
| NGO1061 YP_208143.1 | Putative succinate semialdehyde dehydrogenase | 1.68 |  | COG1012(C) |
| NGO1068 YP_208150.1 | MafB-like protein | 1.80 | 4.78 | NF |
| NGO1070 YP_208152.1 | Hypothetical protein | 2.36 |  | NF |
| NGO1083 YP_208162.1 | Hypothetical protein | 1.58 |  | NF |
| NGO1141 YP_208220.1 | Phage associated protein | 2.82 |  | NF |
| NGO1148 YP_208227.1 | Hypothetical protein | 1.96 |  | NF |
| NGO1163 YP_208241.1 | Hypothetical protein | 1.61 |  | NF |
| NGO1183 YP_208255.1 | Phosphoribosylformylglycinamidine synthase | 1.70 |  | COG0046/COG0047 (F) |
| NGO1234 YP_208305.1 | Hypothetical protein | 1.98 |  | COG1359 (S) |
| NGO1235 YP_208306.1 | Putative acetolactatesynthase isozyme III small subunit IlvH | 1.68 |  | COG0440 (E) |
| NGO1240 YP_208311.1 | Histidinol dehydrogenase HisD | 1.73 |  | COG0141 (E) |
| NGO1241 YP_208312.1 | Histidinol-phosphate aminotransferase HisC | 2.03 |  | COG0079 (E) |
| NGO1242 YP_208313.1 | Imidazoleglycerol-phosphate dehydratase HisB | 2.01 |  | COG0131 (E) |
| NGO1251 YP_208322.1 | Hypothetical protein | 1.80 |  | NF |
| NGO1282 YP_208348.1 | Hypothetical protein | 1.63 |  | NF |
| NGO1283 YP_208349.1 | Phosphoserine aminotransferase | 2.05 |  | COG1932 (HE) |
| NGO1290 YP_208356.1 | Putative amino-acid transporter, sodium/alanine symporter | 2.16 |  | COG1115 (E) |
| NGO1301 YP_208367.1 | Hypothetical protein | 1.71 |  | NF |
| NGO1309 YP_208373.1 | DNA-directed RNA polymerase subunit omega | 1.53 |  | COG1758 (K) |
| NGO1347 YP_208409.1 | Predicted membrane protein | 1.83 |  | COG4648 (S) |
| NGO1368 YP_208428.1 | Antibiotic resistance efflux pump component MtrF | 1.64 | 3.37 | COG2978 (H) |
| NGO1375 YP_208435.1 | Hypothetical protein/ Predicted protease | 1.58 |  | COG3975 R |
| NGO1384 YP_208444.1 | Hypothetical protein | 1.52 |  | NF |
| NGO1387 YP_208447.1 | Hypothetical protein | 1.73 |  | NF |
| NGO1388 YP_208448.1 | Hypothetical protein | 1.67 |  | NF |
| NGO1390 YP_208450.1 | Hypothetical protein | 1.57 |  | NF |
| NGO1405 YP_208462.1 | Hypothetical protein | 2.03 |  | NF |
| NGO1406 YP_208463.1 | Aminomethyltransferase GcvT | 1.54 |  | COG0404 (E) |
| NGO1417 YP_208473.1 | Na(+)-translocating NADH-quinone reductase subunit E, NqrE | 1.54 |  | COG2209 (C) |
| NGO1435 YP_208490.1 | Cadmium resistance protein (CadB) | 2.30 | 3.79 | COG4300 (P) |
| NGO1470 YP_208522.1 | NAD(P) transhydrogenase subunit alpha PntA | 1.68 |  | COG3288 (C) |
| NGO1474 YP_208526.1 | Putative LysR-family transcriptional regulator | 1.53 |  | COG0583 (K) |
| NGO1481 YP_208531.1 | Malonyl-[acyl-carrier protein) O-methyltransferase | 1.53 |  | COG2226 (H) |
| NGO1483 YP_208533.1 | Putative 8-amino-7-oxononanoate synthase (Biotin biosythesis) | 1.58 |  | COG0156 (H) |
| NGO1484 YP_208534.1 | hypothetical protein | 1.65 | 3.33 | COG1376 (S) |
| NGO1487 YP_208537.1 | Arginine decarboxylase | 1.61 |  | COG1166 (E) |
| NGO1513 YP_208563.1 | outer membrane opacity protein D , OpaD protein | 1.52 | 4.38 | COG3637 (M) |
| NGO1578 YP_208619.1 | Transcriptional regulator CysB-like protein | 1.57 |  | COG0853 (K) |
| NGO1585 YP_208626.1 | MafB-like adhesin | 1.82 | 7.29 | NF |
| NGO1588 YP_208628.1 | Hypothetical protein | 2.53 |  | NF |
| NGO1589 YP_208629.1 | Hypothetical protein/ possibly an alternative C terminus for MafB | 1.86 | 9.3 | NF |
| NGO1590 YP_208630.1 | Hypothetical protein | 2.66 |  | NF |
| NGO1591 YP_208631.1 | Hypothetical protein | 2.11 |  | NF |
| NGO1593 YP_208633.1 | Hypothetical protein | 2.31 |  | NF |
| NGO1642 YP_208682.1 | Phage associated protein | 1.56 |  | NF |
| NGO1676 YP_208713.1 | 50S ribosomal protein L21 RplU | 1.56 |  | COG0261 (J) |
| NGO1692 YP_208728.1 | Hypothetical protein/ Predicted membrane protein | 1.52 |  | COG1981(S) |
| NGO1706 YP_208739.1 | Putative LysR-family transcriptional regulator | 1.62 |  | COG0583 (K) |
| NGO1708 (YP_208741.1) | ATP-dependent DNA helicase DinG | 1.56 |  | COG1199 (KL) |
| NGO1726 YP_208485.1 | Hypothetical protein ComFC | 1.67 |  | COG1040 (R) |
| NGO1740 YP_208773.1 | NADH dehydrogenase subunit L, NuoL | 1.65 |  | COG1009 (CP) |
| NGO1746 YP_208779.1 | Hypothetical protein Nuo | 1.53 |  | COG1894 (C) |
| NGO1805 YP_208835.1 | Hypothetical protein | 1.99 |  | NF |
| NGO1879 YP_208912.1 | Hypothetical protein/ Predicted double-glycine peptidase | 1.66 |  | COG3271 (R) |
| NGO1929 YP_208954.1 | Hypothetical protein | 3.84 |  | NF |
| NGO1966 YP_208988.1 | Hypothetical protein | 1.53 |  | NF |
| NGO1970 YP_208991.1 | Hypothetical protein | 1.76 |  | NF |
| NGO1978 YP_208999.1 | Hypothetical protein/Acetyltransferases, including N-acetylases of ribosomal proteins | 1.58 |  | COG1670 (J) |
| NGO1991 YP_209011.1 | Cytosine-specific DNA methyltransferase M. NgoAI | 1.54 | 3.74 | COG0270 (L) |
| NGO2043 YP_209063.1 | Putative dehydrogenase related protein | 2.11 |  | COG1052 (CHR) |
| NGO2086 YP_209099.1 | Hypothetical protein Hia | 1.71 |  | COG5295 (UW) |
| NGO2127 YP_209137.1 | Cadmium resistance protein (CadD protein) | 1.59 | 4.37 | COG4300 (P) |
| NGO2134 YP_209143.1 | 30S ribosomal protein S21 RpsU | 1.56 |  | COG0828 (J) |
| NGO2170 YP_209175.1 | Hypothetical protein | 1.59 |  | NF |
| NGO2173 YP_209178.1 | 50S ribosomal protein L32 RpmF | 1.58 |  | COG0333 (J) |

| Gene Protein | Function | Microarray analysis (change fold) | qRT-PCR (change fold) | COG number (COG category) |
| --- | --- | --- | --- | --- |
| **down** |  |  |  |  |
| NGO1877 YP_208909.1 | Aspartate carbamoyltransferase PyrB | 0.52 |  | COG0540 (F) |
| NGO0032 YP_207213.1 | Hypothetical protein/ putative molecular chaperone | 0.66 |  | COG1214 (O) |
| NGO0048 YP_207227.1 | Carbamoyl phosphate synthase CarB | 0.53 |  | COG0458 (EF) |
| NGO0062 YP_207238.1 | Formate-tetrahydrofolate ligase | 0.64 |  | COG2759 (F) |
| NGO0072 YP_207247.1 | 4-hydroxy-3-methylbut-2-enyl diphosphate reductase IspH | 0.52 |  | COG0761 (IM) |
| NGO0073 YP_207248.1 | Putative phosphatase | 0.66 |  | COG0546 (R) |
| NGO0080 YP_207253.1 | Hypothetical protein | 0.52 |  | NF |
| NGO0081 YP_207254.1 | Predicted metal-dependent phosphoesterases | 0.66 |  | COG0613 (R) |
| NGO0090 YP_207263.1 | Transcriptional repressor NrdR Negatively regulates transcription of bacterial ribonucleotide reductase *nrd* genes and operons by binding to NrdR-boxes | 0.57 |  | COG1327 (K) |
| NGO0092 YP_207265.1 | 3-dehydroquinate synthase AroB | 0.64 |  | COG0337 (E) |
| NGO0104 YP_207277.1 | Chaperone (Uniprot: tRNA N6-adenosine threonylcarbamoyltransferase TsaD) | 0.42 |  | COG1214 (O) |
| NGO0118 YP_207290.1 | N-acetyl-gamma-glutamyl-phosphate reductase ArgC | 0.64 |  | COG0002 (E) |
| NGO0121 YP_207293.1 | Hypothetical protein | 0.67 |  | COG2850 (S) |
| NGO0151 YP_207321.1 | tRNA-dihydrouridine synthase | 0.62 |  | COG0042(J) |
| NGO0158 YP_207327.1 | Aminopeptidase PepN | 0.61 |  | COG0308(E) |
| NGO0179 YP_207346.1 | Hypothetical protein | 0.64 |  | COG3308 (S) |
| NGO0200 YP_207365.1 | Phosphoenolpyruvate synthase PpsA | 0.56 |  | COG0574 (G) |
| NGO0211 YP_207376.1 | Imidazole glycerol phosphate synthase subunit HisF | 0.65 |  | COG0107 (E) |
| NGO0223 YP_207388.1 | Inorganic pyrophosphatase Ppa | 0.58 |  | COG0221 (C) |
| NGO0231 YP_207396.1 | Diadenosine tetraphosphatase, ApaH | 0.65 |  | COG0639 (T) |
| NGO0249 YP_207414.1 | Acetyl-CoA carboxylase subunit beta AccD | 0.64 |  | COG0777 (I) |
| NGO0258 YP_207421.1 | Hypothetical protein | 0.52 |  | COG4859 (S) |
| NGO0274 YP_207436.1 | Tryptophan synthase subunit beta TrpB | 0.45 |  | COG0133 (E) |
| NGO0283 YP_207444.1 | DNA polymerase III subunit delta, HolA | 0.66 |  | COG1466 (L) |
| NGO0378 YP_207534.1 | Hypothetical protein | 0.61 |  | NF |
| NGO0418 YP_207573.1 | Glycosyl transferase family protein RfaG | 0.64 |  | COG0438 (M) |
| Ngo0440 YP_207593.1 | 4-diphosphocytidyl-2-C-methyl-D-erythritol kinase, Ipk | 0.56 |  | COG1947 (I) |
| NGO0473 YP_207623.1 | Phage associated protein | 0.50 |  | NF |
| NGO0486 YP_207636.1 | Phage associated protein | 0.44 |  | NF |
| NGO0488 YP_207638.1 | Phage associated protein | 0.66 |  | NF |
| NGO0498 YP_207648.1 | Hypothetical protein | 0.40 |  | NF |
| NGO0507 YP_207657.1 | Hypothetical protein | 0.65 |  | COG5449 (S) |
| NGO0508 YP_207658.1 | Hypothetical protein | 0.61 |  | COG0791 (M) |
| NGO0512 YP_207662.1 | Phage associated protein | 0.46 |  | NF |
| NGO0517 YP_207667.1 | PemI-like protein (PemI), phage associated protein | 0.58 |  | COG2336 (T) |
| NGO0525 YP_207675.1 | Hypothetical protein | 0.61 |  | NF |
| NGO0566 YP_207711.1 | Prolyl-tRNA synthetase ProS | 0.61 |  | COG0442 (J) |
| NGO0575 YP_207720.1 | tRNA (guanine-N(7)-)-methyltransferase, TrmB | 0.60 |  | COG0220 (R) |
| NGO0587 YP_207730.1 | Hypothetical protein | 0.57 |  | NF |
| NGO0588 YP_207731.1 | Hypothetical protein | 0.58 |  | NF |
| NGO0616 YP_207756.1 | Cell division protein FtsB | 0.62 |  | COG2919 (D) |
| NGO0683 YP_207822.1 | Hypothetical protein | 0.62 |  | COG2340 (S) |
| NGO0684 YP_207823.1 | GTP binding protein HflX | 0.63 |  | COG2262 (R) |
| NGO0704 YP_207840.1 | Bifunctional 3,4-dihydroxy-2-butanone 4-phosphate synthase/GTP cyclohydrolase II-like protein RibB | 0.65 |  | COG0108 (H) |
| NGO0714 YP_207846.1 | Phosphogluconate dehydratase IlvD | 0.53 |  | COG0129 (EG) |
| NGO0740 YP_207869.1 | 3-dehydroquinate dehydratase, AroD | 0.56 |  | COG0710 (E) |
| NGO0759 YP_207886.1 | Hypothetical protein | 0.62 |  | NF |
| NGO0762 YP_207889.1 | Hypothetical protein | 0.53 |  | NF |
| NGO0797 YP_207923.1 | Putative transcriptional regulator HipB | 0.52 |  | COG1396 (K) |
| NGO0829 YP_207951.1 | Chaperone protein HscA homolog, | 0.59 |  | COG0443 (O) |
| NGO0831 YP_207953.1 | Oxidoreductase | 0.57 |  | COG2907 (R) |
| NGO0836 YP_207957.1 | Hypothetical protein | 0.37 |  | NF |
| NGO0848 YP_207965.1 | 2-isopropylmalate synthase LeuA | 0.38 |  | COG0119 (E) |
| NGO0853 YP_207969.1 | Camphor resistance protein/putative fluoride ion transporter CrcB | 0.63 | 0.32 | COG0239 (D) |
| NGO0887 YP_207998.1 | Hypothetical protein | 0.44 |  | COG2128 (S) |
| NGO0934 YP_208042.1 | Hypothetical protein SmtA | 0.66 |  | COG0500 (QR) |
| NGO0953 YP_208055.1 | Hypothetical protein | 0.62 |  | NF |
| NGO0969 YP_208067.1 | Hypothetical protein | 0.66 |  | COG1525 (L) |
| NGO0978 YP_208076.1 | Thiol:disulfide interchange protein DsbD, DipZ | 0.65 |  | COG4232 (OC) |
| NGO0982 YP_208080.1 | Hypothetical protein | 0.42 |  | NF |
| NGO1000 YP_208095.1 | Phage associated protein/ CRISPR/Cas system-associated protein Cas4 | 0.55 |  | NF |
| NGO1034 YP_208122.1 | Hypothetical protein | 0.44 |  | NF |
| NGO1037 YP_208123.1 | Hypothetical protein | 0.55 |  | NF |
| NGO1065 YP_208147.1 | Hypothetical protein | 0.61 |  | COG2879 (S) |
| NGO1097 YP_208176.1 | Hypothetical protein | 0.52 |  | NF |
| NGO1119 YP_208198.1 | Phage associated protein | 0.65 |  | NF |
| NGO1188 YP_208260.1 | Magnesium transporter MgtE | 0.55 |  | COG2239 (P) |
| NGO1286 YP_208352.1 | Translation initiation factor IF-2, InfB | 0.54 |  | COG0532 (J) |
| NGO1293 YP_208359.1 | Hypothetical protein | 0.64 |  | NF |
| NGO1306 YP_208370.1 | Hypothetical protein | 0.63 |  | NF |
| NGO1313 YP_208377.1 | Hypothetical protein | 0.59 |  | NF |
| NGO1331 YP_208393.1 | Chorismate synthase AroC | 0.63 |  | COG0082 (E) |
| NGO1333 YP_208395.1 | DNA topoisomerase IV subunit B GyrB | 0.52 |  | COG0187 (L) |
| NGO1396 YP_208456.1 | Electron transfer flavoprotein-ubiquinone oxidoreductase FixC | 0.44 |  | COG0644 (C) |
| NGO1429 YP_208484.1 | molecular chaperone DnaK (HSP70) | 0.51 |  | COG0443(O) |
| NGO1430 YP_208485.1 | Hypothetical protein | 0.61 |  | NF |
| NGO1444 YP_208498.1 | Hypothetical protein | 0.46 |  | NF |
| NGO1450 YP_208504.1 | Hypothetical protein | 0.63 |  | COG4391 (S) |
| NGO1451 YP_208505.1 | Hypothetical protein | 0.49 |  | NF |
| NGO1493 YP_208543.1 | 30S ribosomal protein S20, RpsT | 0.48 |  | COG0268 (J) |
| NGO1506 YP_208556.1 | NTP pyrophosphohydrolase (MutT family) | 0.44 | 0.59 | COG0494 (LR) |
| NGO1509 YP_208559.1 | DNA repair protein RecO | 0.62 |  | COG1381 (L) |
| NGO1516 YP_208566.1 | Hypothetical protein NnrS | 0.43 |  | COG3213 (P) |
| NGO1519 YP_208569.1 | Hypothetical protein | 0.58 |  | COG1959 (K) |
| NGO1559 YP_208604.1 | hypothetical protein/ outer membrane protein homolog/ Flagellar motor protein MotB | 0.64 | 0.53 | COG2885 (M) |
| NGO1564 YP_208609.1 | Hypothetical protein | 0.44 |  | NF |
| NGO1565 YP_208610.1 | Nicotinate-nucleotide pyrophosphorylase NadC | 0.37 |  | COG0157 (H) |
| NGO1567 YP_208612.1 | Quinolinate synthetase NadA | 0.63 |  | COG0379 (H) |
| NGO1568 YP_208613.1 | L-aspartate oxidase NadB | 0.65 |  | COG0029 (H) |
| NGO1576 YP_208617.1 | Hypothetical protein | 0.56 |  | NF |
| NGO1664 YP_208701.1 | Hypothetical protein | 0.61 |  | NF |
| NGO1668 YP_208705.1 | Glucose-6-phosphate isomerase, Pgi | 0.65 |  | COG0166 (G) |
| NGO1671 YP_208708.1 | Dephospho-CoA kinase CoaE | 0.48 |  | COG0237(H) |
| NGO1679 YP_208716.1 | 50S ribosomal protein L33, RpmG | 0.60 |  | COG0267 (J) |
| NGO1686 YP_208723.1 | Hypothetical protein NlpD | 0.60 |  | COG0739 (M) |
| NGO1733 YP_208766.1 | Putative ribosome biogenesis GTPase RsgA | 0.65 |  | COG1162 (R) |
| NGO1771 YP_208802.1 | Hypothetical protein MscS | 0.37 |  | COG0668 (M) |
| NGO1775 YP_208806.1 | Ferredoxin 2 | 0.49 | 0.51 | COG2878 (C) |
| NGO1783 YP_208814.1 | Pseudouridine synthase protein RluC | 0.48 |  | COG0564 (J) |
| NGO1785 YP_208816.1 | Ribonuclease E CafA | 0.65 |  | COG1530 (J) |
| NGO1793 YP_208824.1 | Hypothetical protein | 0.56 |  | COG1289 (S) |
| NGO1866 YP_208898.1 | Two-component system transcriptional response regulator AtoC | 0.55 |  | COG2204 (T) |
| NGO1867 YP_208899.1 | Two-component system sensor kinase NtrY | 0.64 |  | COG5000 (T) |
| NGO1868 YP_208900.1 | Hypothetical protein | 0.64 |  | NF |
| NGO1871 YP_208903.1 | Peptide deformylase Def | 0.63 |  | COG0242(J) |
| NGO1876 YP_208908.1 | Aspartate carbamoyltransferase PyrI | 0.61 |  | COG1781(F) |
| NGO1878 YP_208910.1 | Hypothetical protein RimI | 0.50 |  | COG0456 (R) |
| NGO18781 YP_208911.1 | Hypothetical protein | 0.20 |  | NF |
| NGO1889 YP_208918.1 | Hypothetical protein | 0.61 |  | COG3755 (S) |
| NGO1895 YP_208923.1 | Hypothetical protein Tex | 0.65 |  | COG2183 (K) |
| NGO1907 YP_208933.1 | Hypothetical protein | 0.66 |  | COG0325 (R) |
| NGO1952 YP_208975.1 | Hypothetical protein | 0.20 |  | NF |
| NGO1959 YP_208982.1 | Hypothetical protein | 0.59 |  | NF |
| NGO2004 YP_209024.1 | Hypothetical protein | 0.61 |  | NF |
| NGO2010 YP_209030.1 | Putative TldD protein | 0.66 |  | COG0312 (R) |
| NGO2033 YP_209053.1 | Hydrolase GloB | 0.55 |  | COG0491 (R) |
| NGO2077 YP_209090.1 | Putative gluconate permease, GntP protein | 0.65 |  | COG2610 (GE) |
| NGO2093 YP_209106.1 | Ferric enterobactin receptor CirA, FetA | 0.48 | 0.51 | COG1629 (P) |
| NGO2125 YP_209135.1 | Putative acetyltransferase WbbJ | 0.65 |  | COG0110 (R) |
| NGO2133 YP_209142.1 | Hypothetical protein | 0.64 |  | COG1610 (S) |
| NGO2154 YP_209162.1 | Glycine--tRNA ligase beta subunit, GlyS | 0.51 |  | COG0751 (J) |
| NGO2162 YP_209168.1 | Hypothetical protein | 0.47 |  | COG3012 (S) |
| NGO2182 YP_209187.1 | 50S ribosomal protein L34, RpmH | 0.63 |  | COG0230 (J) |
